# Overview of junctional complexes during mammalian early embryonic development

**DOI:** 10.3389/fendo.2023.1150017

**Published:** 2023-04-20

**Authors:** Ceren Canse, Ecem Yildirim, Aylin Yaba

**Affiliations:** ^1^ Faculty of Medicine, Yeditepe University, Istanbul, Türkiye; ^2^ Department of Histology and Embryology, Yeditepe University Faculty of Medicine, Istanbul, Türkiye

**Keywords:** junctional complexes in preimplantation embryo development, adherens junction, desmosome, tight junction, gap junction, preimplantation embryo

## Abstract

Cell-cell junctions form strong intercellular connections and mediate communication between blastomeres during preimplantation embryonic development and thus are crucial for cell integrity, polarity, cell fate specification and morphogenesis. Together with cell adhesion molecules and cytoskeletal elements, intercellular junctions orchestrate mechanotransduction, morphokinetics and signaling networks during the development of early embryos. This review focuses on the structure, organization, function and expressional pattern of the cell–cell junction complexes during early embryonic development. Understanding the importance of dynamic junction formation and maturation processes will shed light on the molecular mechanism behind developmental abnormalities of early embryos during the preimplantation period.

## 1 Introduction

**Figure 1:**
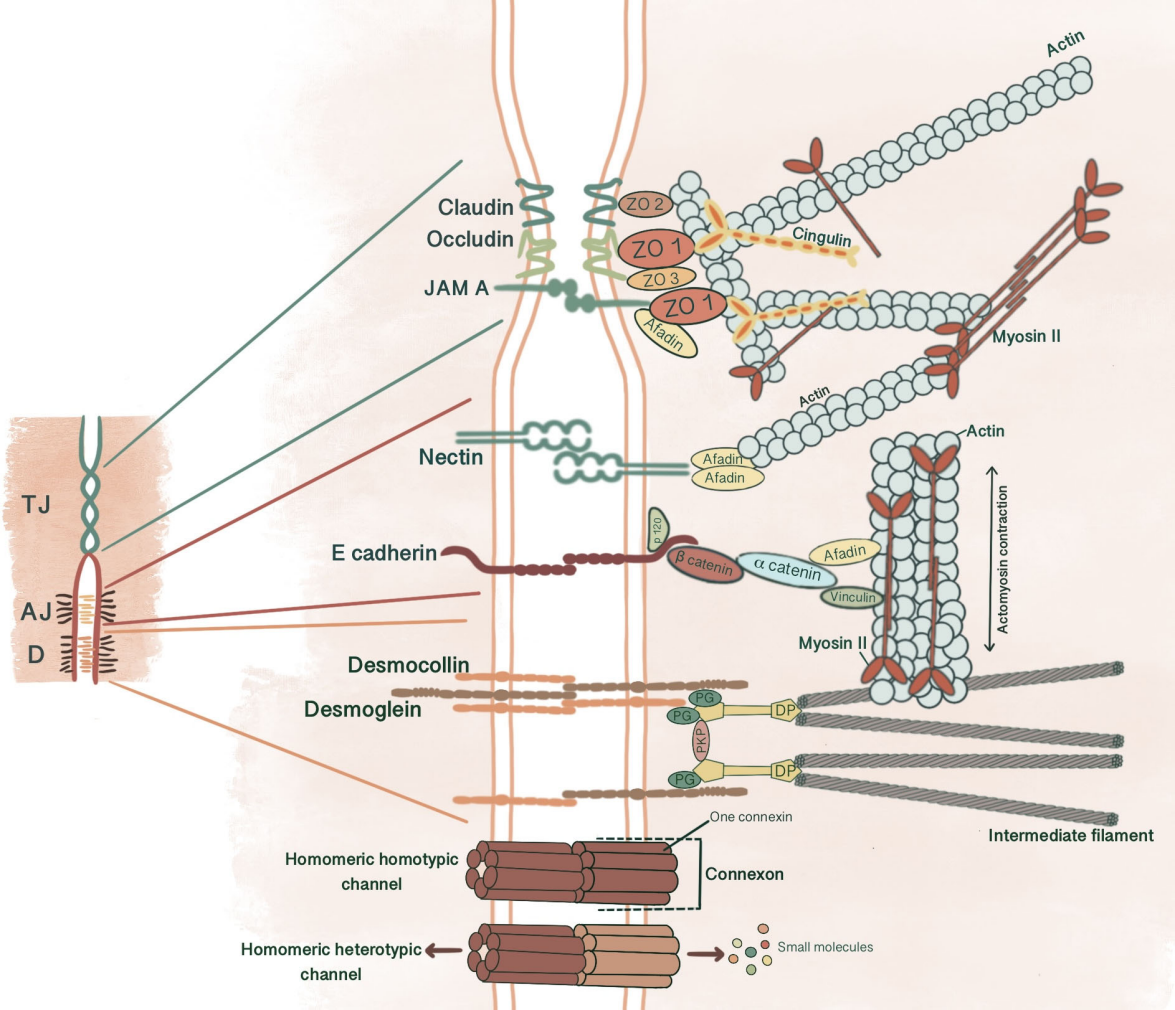
Simplified schematic presentation of basic structural components and molecular composition of intercellular junctions. (TJ, Tight junctions; AJ, Adherens junctions; D, Desmosomes; ZO, Zonula occludens; PG, Plakoglobin, PKP, Plakophilin; DP, Desmoplakin).

Successful implantation of an embryo into the endometrium requires the specification of extra-embryonic and embryonic lineages that will give rise to the placenta and the embryo itself. As a result of cleavage, compaction, radial polarization and asymmetric divisions of the newly formed zygote, two cell lineages of different developmental potential arise with an external outer cell layer, the trophectoderm, possessing epithelial characteristics enclosing a population of non-epithelial cells, the inner cell mass (ICM) (1). Epithelial cells are characterized by an apical membrane confronting the external environment, lateral membranes contacting neighboring cells and basal domains anchored to a basement membrane interacting with the extracellular matrix (1). This kind of organization subdivides cells, and thus tissues, morphologically and functionally into different compartments, enabling maintenance of an ion- and size-selective diffusion barrier, cell shape, cellular adhesion, communication, and cytoplasmic/surface polarity, which are all essential for intracellular machinery (2–4). Likewise, the outer trophectodermal cell layer, an epithelium analogue, serves as a chemical barrier that separates the embryo from the external uterine environment, maintaining blastocyst integrity, developmental potential and viability. This review focused on the epithelial characterization of the pre-implantation embryo and the structure, biogenesis and developmental functions of intercellular junctions that are essential for developing such phenotype. Functionally, intercellular junctions can be classified into three categories: 1-Anchoring junctions divided into adherens junctions (AJ), desmosomes, hemidesmosomes and focal adhesions; 2-Tight junctions (TJ), and 3-Gap junctions (GJ) (4) (**Figure 1**). Individual roles of all of these junction types concerning trophectoderm maturation and key events of embryo development will be emphasized separately. By assembling our current understanding of junction biogenesis during embryonic development, we aim to support studies on assisted reproductive technologies and embryo selection for IVF.

## 2 Adherens junctions

AJs are cell-cell adhesion complexes that form extracellular adhesive contacts between cells to maintain tissue cohesion, sense and respond to tensile forces at the contact interface, establish cell polarity and form intracellular links to cytoskeletal elements (5, 6). AJ’s ability to localize proteins to subcellular compartments allows modulation of signaling pathways (7). AJs comprise three main components: transmembrane cadherins, armadillo family members and cytoskeletal adaptor proteins. This core cadherin-catenin complex binds to actomyosin cytoskeleton and signaling proteins, influencing the overall mechanobiology of cells.

Depending on the tissue types and developmental stages, different AJ conformations might be present along the cell-cell interface. These variants of AJ such as linear AJ, focal AJ, zonula AJ (ZA), tricellular AJ and fascia AJ differ in their molecular composition, organization of the associated actomyosin skeleton and stability (6). Newly forming AJs are discontinuous cellular adhesions, characterized by a spot-like, punctate appearance. In maturing epithelial cells, the apical junctional complex contains ZA junctions underneath the apical TJ, forming a tight belt-like structure that links cells into continuous sheets, creating highly polarized cells with separate apical and basolateral membranes (6, 8).

Cadherins initiate cellular adhesion through forming trans-dimers with adjacent cadherins *via* their most distal extracellular domain in a calcium-dependent manner (6). The cadherin family consists of type 1 cadherins (e.g. E- cadherin expressed broadly in epithelia, P-, N-, M-, R-cadherin), type 2 cadherins (e.g. VE-cadherin restricted to vasculature), desmosomal cadherins (desmocollin and desmoglein) and subfamily of cadherin-like molecules (9). E-cadherin, a type of classical cadherin, is a single-pass transmembrane protein comprising five extracellular cadherin domains which are bound together by Ca^+2^ ions, a transmembrane domain and a C-terminal cytoplasmic domain (6, 10). The intracellular domain consists of a juxtamembrane domain (JDM) that binds p120-catenin and α-catenin binding domain (CBD) which binds β-catenin (5, 10). Catenins are cytoplasmic proteins that allow interaction of cadherin complex with the cytoskeletal elements. Catenins contribute to cadherin function in three ways: enabling direct physical link of cadherins to actin cytoskeleton, regulating signaling to cytoskeleton by tyrosine kinases and small GTPases, controlling the adhesive state of the cadherin extracellular binding domain (11). β-catenin consists of an amino-terminal region, a central domain of 12 armadillo arm repeats and a carboxy-terminal (10). p120-catenin contains 9 arm repeats preceded by an amino terminal sequence that varies in length, creating four splice varients (10). p120 catenin association with E-cadherin is essential in the formation of stable cell-cell adhesions. In cells expressing p120-uncoupled E-cadherin, there is a failure in the formation of continuous circumferential actin ring and insertion of these actin cables into peripheral concentrations of E-cadherin. This causes a failure in transit from loose to tighter cell-cell adhesions (12). α-catenin, the link between the cadherin-catenin complex and the cytoskeleton, contains 1) an N-terminal domain that binds β-catenin and plakoglobin 2) a central modulatory domain that binds vinculin, α -actinin; 3) a C-terminal domain that binds ZO-1, F-actin and α1-helix. Thus, α-catenin acts as the mechanosensor and mechanotransducer that goes under conformational changes in response to force and regulates actin cytoskeleton in a tension-dependent manner (6, 11). In addition, α-catenin is responsible in maintenance of junction stability. α-catenin suppressed cells lose their cell-cell contacts and demonstrate disrupted localization of β-catenin, E-cadherin and TJ protein ZO-1 (13) (**Figure 1**).

E-cadherin, α-catenin and β-catenin mRNA are all present in unfertilized eggs, 2-cell, 8-cell and blastocyst stages, with a decrease at the 2-cell stage. Maternally derived E-cadherin mRNA and protein is present in unfertilized egg and 1 cell-stage embryo, assembled into a protein complex with catenins to enable adhesive interactions between oocyte and cumulus cells (14). However, *de novo* E-cadherin transcription from the embryonic genome starts at the late 2-cell stage. With immunoelectron microscopy, 2- and 4-cell stage embryos demonstrate an even distribution of E-cadherin on cell surface of blastomeres. At the 4-cell stage, E-cadherin concentration increases in close membrane appositions. Following the 8-cell stage and compaction, E-cadherin starts accumulating at cell contact sites, removed from the apical membrane domain. In 16- and 32-cell stages, E-cadherin is redistributed in cells committed for epithelial cell differentiation and becomes basolaterally localized between adjacent cells in the outer cells of the morula and TE. Whereas, inner cells and ICM demonstrate an even distribution along the cell membrane (15). LIMK is essential for early cleavage compaction through AJ assembly and actin filament organization. LIMK1/2 knockdown in porcine embryos causes abnormal cleavage, reduced blastocyst formation, disrupted localization of β-catenin, E-cadherin, ZO-1, CXADR and decreased cortical actin levels (16). The basolaterally distributed E-cadherin associates with catenins that in turn connect with cytoskeletal structures. This cytoskeletal anchorage of the cadherin-catenin complex to actin influences the strength of adhesiveness of E-cadherin (17). With immunofluorescence microscopy, E-cadherin-catenin complex demonstrates a strong membranous localization at cell-cell contact sites in all development stages of mouse embryo, including 2-cell stage. (18). Rac 1, a small GTPase, is localized adjacent to cell membranes of 2- and 4- cell stage blastomeres and shifts to the cytoplasm with compaction. Additionally, Natale et al. Suggested that Rac-1 is an important regulators of E-cadherin-catenin complex during murine preimplantation embryonal development (19). β-catenin is detected in the surface and the pronuclei of zygotes, whereas at the surface of blastomeres of 2- and 8-cell embryos. (20). So, at the 2-cell stage, these adhesion proteins already form a complex and are localized to cytoskeleton-bound membrane domains to enable cell-cell contact between blastomeres. In immunofluorescence evaluation of the mouse embryos, total and active (dephospho) β-catenin is expressed in all stages of preimplantation development, starting from the 1-cell stage to blastocyst. While before the morula stage, active β-catenin is mostly localized in nuclei of all embryonic cells, it is predominantly present in TE of blastocysts (21). After the hatching of fully expanded blastocyst from zona pellucida for implantation, the signal for active β-catenin disappears. On E6, β-catenin appears in invasive trophoblasts, followed by the embryo on day 7 (22). In human embryos, β-catenin protein is localized in the cortical region under the cell membrane at 6-cell, 8-cell stage embryos, and both TE and ICM of blastocysts (23).

E-cadherin ^(-/-)^ embryos show defects in molecular architecture involving ZA, TJ and cortical actin filament organization. In addition, α- and β-catenin expression levels are significantly reduced (24). Removal of the N-terminal part of β-catenin, that allows the binding of α-catenin and E-cadherin, in oocyte disrupts adhesion between individual blastomeres. In addition, E-cadherin mislocalizes to the cytoplasm in 2- and 4-cell stage embryos, which is reversed during 4- to 8- cell stage transition with paternal allele synthesis of β-catenin (20).

### 2.1 E-cadherin

There are four prominent roles of E-cadherin containing ZA in pre-implantation embryonic development: 1) Mediating compaction, 2) Triggering radial polarization by inducing PAR asymmetry *via* cell contact cues, 3) Cell fate determination by differential activation of the Hippo signaling pathway in inner and outer cells through E-cadherin & apical polarity complex association with AMOT, 4) Directing hydraulic fracturing during blastocoel formation by reorganization of E-cadherin at cell-cell contacts.

#### 2.1.1 Mediating compaction

Embryo compaction, which is the first critical morphogenetic process that occurs during embryonic development is initiated at the 8-cell stage (25). Starting from cell contact points proceeding outwards, adjacent blastomeres flatten against each other, maximizing their contacts and generating a mass of cells with no distinguishable cell boundaries (26). Although the exact mechanism that drives compaction is still unknown, three primary processes were suggested to mediate this morphological change: increased cell-cell contact due to a change in cellular adhesive properties, E-cadherin dependent filopodia formation and actomyosin mediated increase in surface contractility (27). AJ links to the cell cytoskeleton elements like actin, myosin 1 and ezrin/radixin/moesin is essential in mediating cell shape changes during compaction by transmitting the main driving forces generated by the actin cytoskeleton to the cell surface (27). Ca^+2^ dependent cell-cell adhesion system generates the force necessary for early 8-cell compaction. E-cadherin redistributes contractility generated by actomyosin away from cell-cell contact points toward the cell surface, causing a twofold increase in tension at the cell-medium interface (28). When embryos are cultured in Ca^+2^ free medium, compaction is inhibited. In addition, when embryos that have already compacted *in vivo* are transferred to Ca^+2^-free medium, embryos uncompact within 30 minutes (26).

Embryos homozygously knockout for E-cadherin, although undergo compaction due to residual maternal E-cadherin, fail to form blastocyst cavity and show prenatal lethality (29). Embryos lacking maternal E-cadherin show a lack of adhesion in blastomeres until the late morula stage, when E-cadherin translated from paternal alleles reaches the blastomere surface. Compaction of embryos that lack maternal E-cadherin is enabled, although a cell division later than normal embryos, by adequate blastomere contact with confinement within the zona pellucida until paternal E-cadherin is synthesized (20). In the absence of both maternal and zygotic E-cadherin, embryos develop as loose aggregates of cells, never compact and do not show normal epithelial morphology in outer blastomeres (30).

Live mouse embryo imaging demonstrates filopodia containing E-cadherin, F-actin and Myo 1 extending onto neighboring cells, transmitting the cell-autonomously generated cortical tension. There is temporal coordination between filopodia extension-retraction and cell shape change. Cells that are relatively round before filopodia extension undergo elongation at their apical border and become rounded again before division by filopodia retraction. Knockdown of filopodia components such as E-cadherin, α- and β-catenin causes failure in compaction. Comparably, inducing filopodia formation by increased expression of Myo10 causes premature compaction of the embryo (31).

#### 2.1.2 Triggering radial polarization by inducing PAR asymmetry via cell contact cues

**Figure 2:**
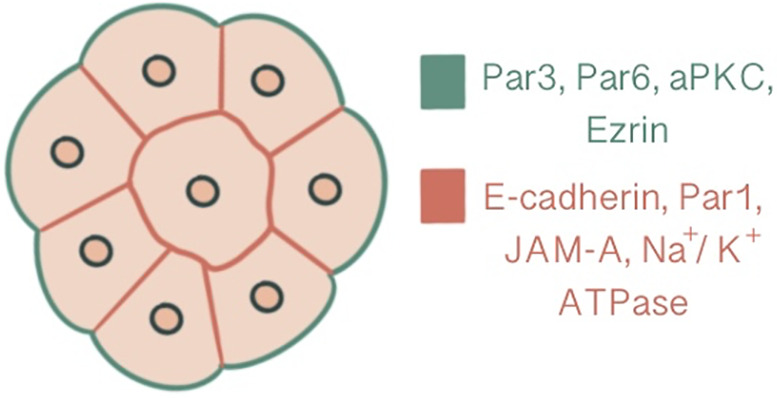
Polarization demonstrated at a 16-cell stage embryo, central slice. Ezrin, Par6, Par3, aPKC are present at the apical domain which is enriched with actin, whereas Par-1, JAM-A, Na/K ATPase and E-cadherin are localized at the basolateral domain.

In parallel to compaction, radial polarity, the asymmetric localization of apical and basolateral polarity regulators, develops at the 8-cell stage as the second key morphogenetic event of the mouse embryonic development (1). The cell contact region where the junctional proteins will be recruited comprises the basolateral domain. In contrast, the contact-free surface enriched with microvilli, actin and actin-binding proteins marks the apical domain (32). The majority of cells become polarized in 6 hours within the generation of an 8-cell embryo, while some may start as early as 2 hours. The presence of cell contact is required to generate polarity, as the contact points guide the axis of polarization (33, 34). This polarity axis organizes cytoplasmic content and serves as a memory for generating two distinct cell types with differentially inherited polarized cellular domains (33). Subcellular machinery of specification of the polarized cell surfaces in embryos is regulated by spatial and temporal localization of apical and basolateral polarity proteins (35). Three main polarity regulator proteins ultimately scaffold Rho GTPase to specific membrane domains: the Par group at close localization to AJC and the primary cilium, the crumbs complex at the apical side of AJC and the scribble complex on lateral membranes below the AJC (1, 3, 36). Par3-Par6-aPKC is the core complex that regulates polarity in pre-implantation embryos. Par-6 binds to the amino terminus of Par-3 through its PDZ-1 domain. Par-6 associates with aPKC *via* direct head-to-head association and forms a complex that can associate with and phosphorylate Par-3 (37, 38). E cadherin is one of the first proteins to demonstrate a polarized distribution at cell contact sites with compaction, followed by Ezrin, Par6, Par3, aPKC at apical domain and Par-1, JAM-1, Na/K ATPase at basolateral domain (1, 39) (**Figure 2**). E-cadherin mutant embryos demonstrate an overlap between apical and basolateral proteins: PKCζ becomes localized throughout the majority of outer cell membrane, overlapping with Na/K ATPase β-1 subunit, Jam1 and Lgl1. In addition basolateral markers also accumulate as intracellular puncta and vacuole-like structures within the cell (30).

In the mouse embryo, *de novo* polarization follows two steps: 1) Actomyosin localization to the cell-contact free surface during the early-to-mid 8-cell stage by PKC-RhoA activation through PLC-mediated PIP2 hydrolysis, 2) Apical enrichment of PAR proteins at mid-late 8-cell stage forming a mature apical cap, whereas F-actin redistribution to form a ring-like structure around the PAR enriched domain (40). Restriction of Par polarity proteins to contact free surface has two distinct effects: a developmental role in cell fate specification by modification of the Hippo signal pathway and an effect on tissue morphogenesis by directing cytoskeletal asymmetries (1, 37).

After polarization and compaction, the morula undergoes two sets of asymmetric divisions (from 8-to-16 cells and 16-to-32 cells), generating two distinct cell populations that occupy different positions within the morula (27, 41–;43). The inheritance of the apical domain, depending to the division angle of the embryo, determines the fate of daughter cells (43). Polar cells, which have an apical surface, become positioned peripherally while apolar cells are placed centrally within the embryo (43). This process of differential inheritance is the foundation of ICM and TE lineage differentiation (43). SPECC1 is detected at apical cell-cell contacts at the blastocyst stage, and knockdown of SPECC1 disrupts paracellular sealing, reducing the rate of blastocyst development (44). Recent studies showed division-independent routes, such as internalization, for inner cell allocation. Samarage et al. suggest that apical constriction of the cortical actomyosin network is the primary morphogenetic mechanism involved in the first spatial segregation of cells. In the 12-cell stage, constricting cuboidal shape daughter cells with high cortical tension decrease their apical surface and gradually position closer to the center of the embryo. Daughter cells demonstrating a lower tensile force remain outside and adopt a wedge shape. Although the junction length between constricting cells and their neighbors decreases, E-cadherin levels or mobility do not differ among cells during apical constriction. Myosin II distribution heterogeneity has a more specialized role in inner cell allocation rather than E-cadherin-mediated cell adhesions (45).

#### 2.1.3 Cell fate determination by differential activation of the Hippo signaling pathway in inner and outer cells through E-cadherin & apical polarity complex association with AMOT

**Figure 3:**
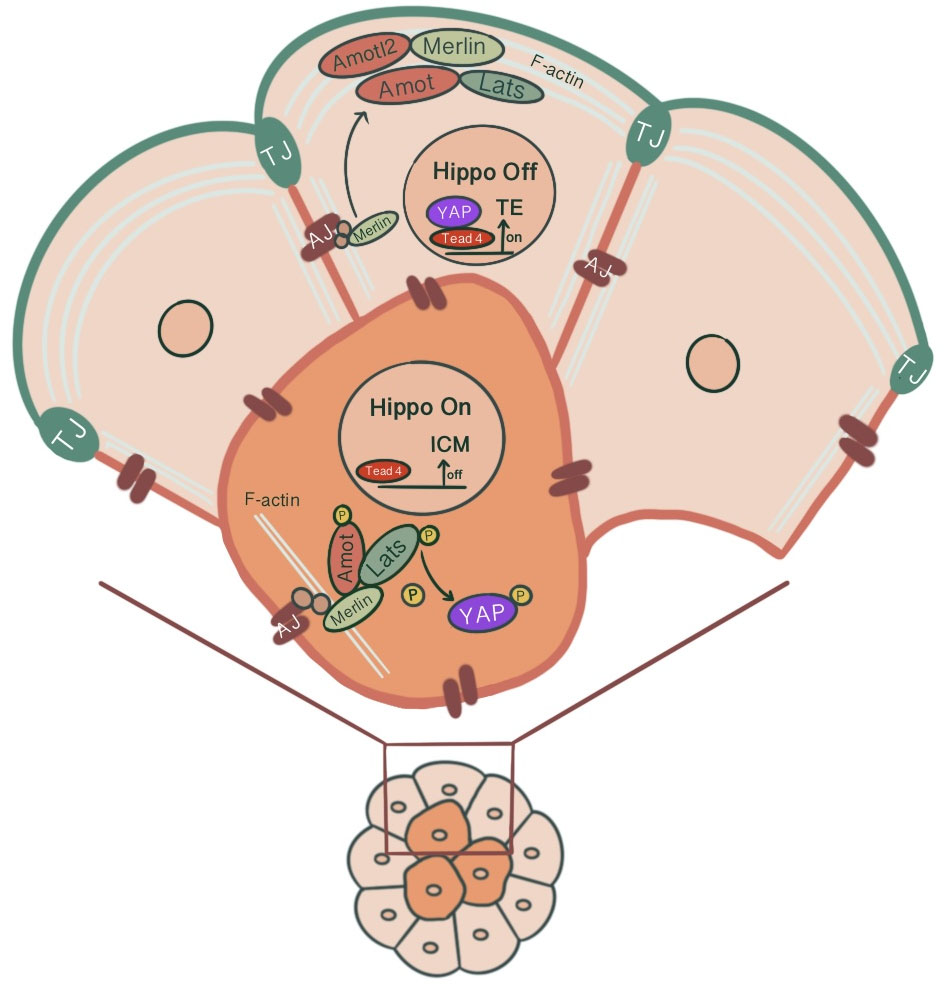
Differential modulation of the Hippo signaling pathway in inner and outer cells of the preimplantation embryo, influenced by junction-associated scaffold protein angiomotin (Amot) and its interaction with tight junctions & adherens junctions. In outer cells, Amot co-localizing with ZO-1 causes apical F-actin-mediated suppression of the Hippo signaling pathway. Through sequestration of Amot to the apical membrane domain *via* the Par-aPKC complex, Yap, which can translocate to the nucleus, causes outer cells to adopt TE fate *via* Tead4 activation and Cdx2 expression. In inner cells, the E-cadherin-β-catenin-α-catenin-Merlin-Amot complex acts as an upstream regulator of the Hippo signaling pathway through Yap sequestration at cytoplasm, repressing target gene expression.

The transition from inner/outer cells to pluripotent ICM/TE lineages is achieved through the control of the Hippo signaling pathway and its effects on lineage-specific gene expressions (46). For TE specification, Caudal-related homeobox 2 transcription factor (Cdx2) expression along with POU-family transcription factor Oct3/4 and Nanog repression is required. Cdx2 expression that starts ubiquitously at the time of polarization progressively increases in outside cells, eventually leading to positive regulation of trophectoderm lineage markers (47). Pluripotent embryonic stem cells show morphological differentiation to TE lineage by overexpression of Cdx2 and similarly by forced repression of ICM-specific transcription Oct4 (48, 49). The differential activation of target genes *via* Hippo signaling in outer/inner cells is achieved by modulation of the activity of transcription factor Tead4 through the differential localization of Tead4 coactivator protein Yap. The inner cells, with the activation of the Hippo pathway, Yap phosphorylation by Lats kinase1/2 sequesters Yap in the cytoplasm, causing Tead4 to remain inactive, repressing target gene expression. However, in the absence of Yap phosphorylation, Yap’s nuclear accumulation and Tead4 activation induce Cdx2 expression in outer cells, promoting TE fate (27, 50) (**Figure 3**). As a result, a blastocyst with two different cell populations is formed.

Intercellular adhesions play a critical role in determining cell fate and establishing a position dependent Hippo signaling through their effect on cell polarity. (7). In E-cadherin mutant embryos, although a blastocoel cavity or TE do not develop, differential expression of Cdx2 and Oct4 is still observed. However, it occurs in a disrupted ratio, with more cells expressing Cdx2 in mutant embryos compared to the wild-type. Cdx2(-/-) embryos fail to maintain blastocoel and epithelial integrity due to disrupted localization of ZO-1 α-, ZO-1 α+ and E-cadherin (51). Thus, although individual cells can initiate ICM- or TE-like fates, E-cadherin is necessary for correctly allocating cell fate and normal spatial distribution of ICM- and TE-like cells (30). Bovine zygotes treated with E-cadherin dsRNA demonstrate significantly lower blastocyst formation rate (52). E-cadherin has a unique function in TE formation. Gene replacement by N-cadherin cDNA introduction into the E-cadherin genomic locus and inactivation of maternal E-cadherin allows compaction and expression of epithelial makers in outer cells. However, a fully polarized epithelium, TE, cannot form due to the failure to correctly assemble junctions and cytocortical networks (53).

It has been suggested that junction-associated scaffold protein angiomotin (Amot) acts as a molecular switch for Hippo signaling pathway. In inner cells, Amot causes activation of Hippo signaling through its interactions with AJs. In contrast, in outer cells, there is an apical F-actin mediated suppression of the Hippo signaling pathway (54). In 16 cell stage, Amot becomes differentially localized among inner/outer cells. While it is localized to the apical membranes of outer cells, inner cells demonstrate a distribution throughout the membranes. This is maintained until the early blastocyst stage. In non-polar inner cells, Amot localizes at AJs through interacting with Merlin by its coiled-coil domain, forming a large complex of E-cadherin-β-catenin-α-catenin-Merlin-Amot, which acts as an upstream regulator of the Hippo signaling pathway. N-terminal of Amot mediates actin binding, Nf2/Merlin mediated interactions with E-cadherin and associations with Lats kinases (55). Phosphorylation of Amot by LATS1/2 kinases suppresses the actin-binding activity of Amot and causes its mislocalization from cortical F-actin in the junctional sites stabilizing it in the basolateral AJs. This adds another layer of regulation to the Hippo signaling pathway where phosphorylation of Amot acts as a switch for phosphorylation and cytoplasmic sequestration of Yap (55–57). Maternal-zygotic Nf2 mutant embryos show defects in Yap localization and Cdx2 expression, where inner cells resemble TE and ultimately lose their normal ICM identity (58). In MDA-MB-231 cell line, it has been shown that doxycycline-induced expression of E-cadherin and homophilic binding of E-cadherin between two adjacent cells cause redistribution of Yap from the nucleus to cytoplasm. Additionally, endogenous depletion of β-catenin in MDA-MB-231 cells cause increased nuclear accumulation of Yap and phosphorylation on the S127 residue (57). Similarly, cell contact inhibition using E-cadherin blocking antibody ECCD1 inhibits the nuclear accumulation of Yap in the inside cells of the morula (50). Correspondingly, Yap knockdown in porcine embryos significantly reduced development to 8-cell and blastocyst stage, TE cell number and increased Cdx2 negative cells. Additionally, decreased gene expression involved with cell fate specification (*Cdx2, Tead4, Oct4, Sox2, Nanog)*, junction assembly (*Ocln, Cldn4, Cldn6, Cldn7, Tjp1, Tjp2, Cdh1*), and fluid accumulation was evident (59) (**Figure 3**). A study showed that knockdown of AP-2γ (Tcfap2c), a novel upstream regulator of *Cdx2* expression, causes downregulation of *Pard6b, Tjp2, Cldn4, Cldn6, Cldn7* and prevents TJ formation and paracellular sealing at apical cell borders, inhibiting blastocyst formation (60, 61). Knockdown of INO80 in porcine embryos impaired blastocyst development, paracellular sealing of TE, and decreased expression of OCT4, CDX2, TEAD4, CDH1, OCLN and several cell polarity, cytoskeleton and fluid-accumulation related genes (62).

In the outer cells, Amot is present at TJs, co-localizing with ZO-1. Par-aPKC complex sequesters Amot to the apical membrane domain, held in an inactive state by interacting with F-actin (54, 55). As a result, unphosphorylated Yap can enter the nucleus. Pard6b, a homolog of the *Par-6* gene, knockdown causes failure in blastocoel cavity formation due to defective TJ formation, abnormal cell polarization, abnormal distribution of actin filaments and, as a result, diminished expression of Cdx2 (63) (**Figure 3**).

#### 2.1.4 Directing hydraulic fracturing during blastocoel formation by reorganization of E-cadherin at cell-cell contacts

**Figure 4:**
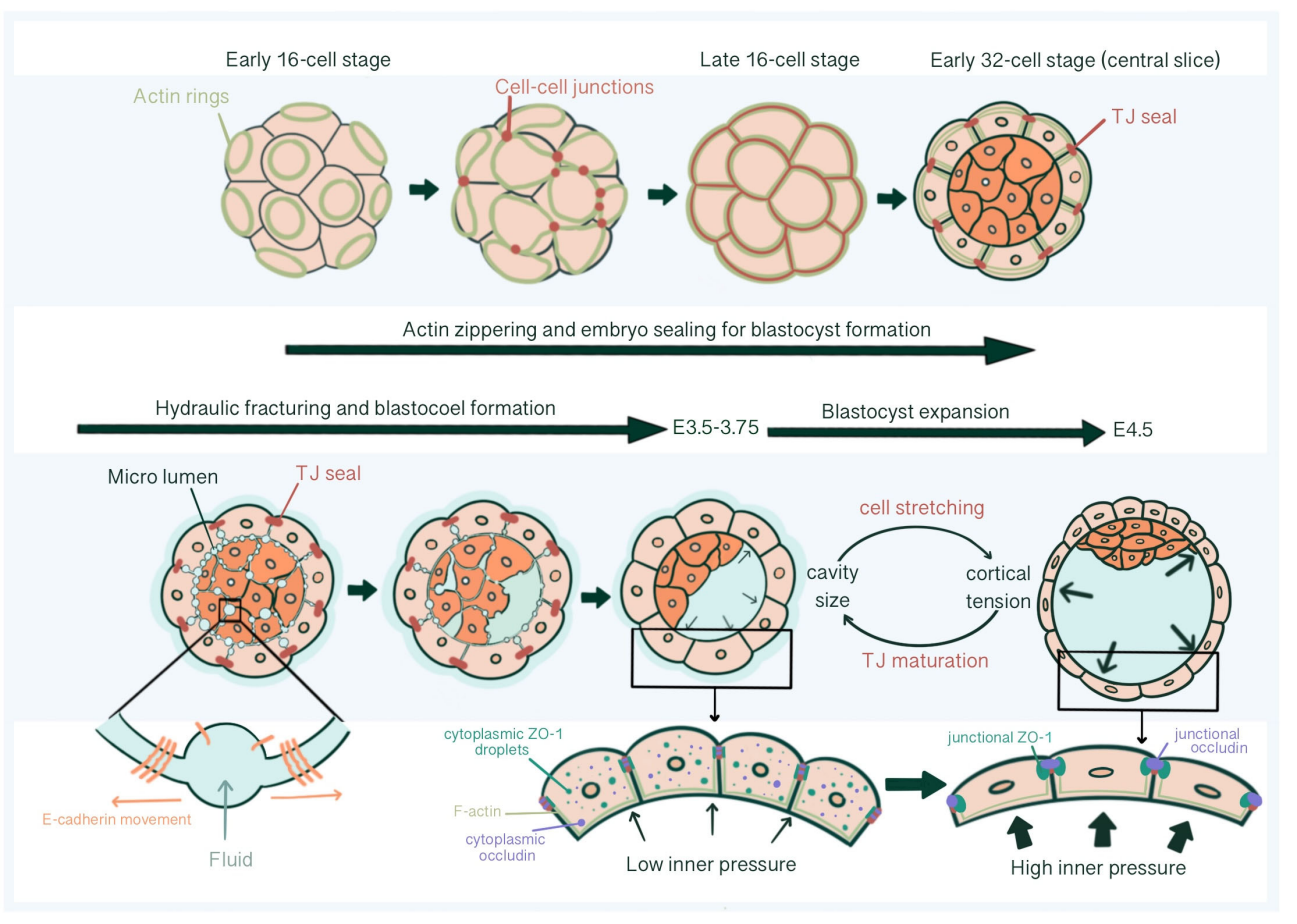
Tight junction seal formation and force dependent junctional maturation, enabling hydraulic fracturing and blastocoel expansion, from E2.75 until E4.5. The actin ring structure formed in the apical region of the blastomeres in the early 16-cell stage embryos affects intercellular junction maturation and formation of TJ sealing. With hydraulic fracturing coordinated *via* reorganization of E-cadherin at cell-cell contacts, fluid accumulates within the embryo forming the blastocoel The integrity of blastocyst depends on mature TJ seals that prevents collapse with increasing inner pressure. Force dependent (increased luminal pressure and cortical tension, accompanied with TE cell stretching) junctional maturation enables accommodation to pressure increase and blastocoel expansion prior to hatching.

Blastocoel cavity formation starts as micro-lumens through a process of hydraulic fracturing. Micro-lumens form at cell-cell contacts causing fluid accumulation from the outside environment into intercellular space regarding an osmotic gradient, established by differential Na^+^ concentration by Na^+^/K^+^ ATPases at the basolateral membrane together with basolateral Aquaporin 3 and 8 mediated trans-cellular water influx (64–67). For this, E-cadherin must be redistributed and accumulate at micro-lumen edges, causing a separation of previously cohesed cell membranes (65). The intercellular connections of micro-lumens allow fluid to move from smaller micro-lumens into larger ones, parallel with the Young-Laplace equation, creating one large, dominant micro-lumen: the blastocoel (66) (**Figure 4**).

### 2.2 β-catenin

β-catenin has a dual function as a transcriptional effector of Wnt signaling and a constituent of AJs (68). Wnt signaling is an important regulator of the maintenance, self-renewal and differentiation of adult mammalian tissue stem cells (69–71). In addition, it has been demonstrated to regulate major embryonic events such as anterior-posterior patterning (72) transcriptional programmes at gastrulation (73) specification of the primitive streak and distal visceral endoderm (68, 64, 75). In the canonical Wnt pathway, β-catenin functions as a transcriptional co-activator. In the absence of Wnt, β-catenin undergoes proteasomal degradation by a destruction complex residing in the cytoplasm, preventing it from reaching the nucleus and interacting with Wnt target genes. When Wnt binds to a heterodimeric receptor complex, consisting of Frizzled receptor and its co-receptor LRP6 or LRP5, Wnt-Fz-LRP6 leads to inhibition of Axin-mediated β-catenin phosphorylation. β-catenin is stabilized and translocated into the nucleus, forming complexes with TCF/LEF and activating Wnt target gene expression (76). Transcripts of ligands and antagonists of the Wnt signaling pathway were detected in the mouse preimplantation embryos. *Wnt3a* and *Wnt -4* start to be detected in 4-cell and precompact 8-cell stage embryos, with robust enhancement in expression with compaction. *Wnt3a* transcripts in 2-cell stage embryos have not been specifically determined whether they are from a maternal or embryonic origin (77). *Wnt-1, -3, -3a, -4, -5a, -5b, -6, -7a, -7b, -9a, -10b*, *-11*, -*13*, and Wnt signaling antagonists *Sfrp1* and *Dkk1* are all present in mouse blastocysts, detected by qRT-PCR (78–82). Amongst, *Wnt-3a, -6, -7b*, -*9a* and *-10b* have the highest gene expression levels (81, 82). With exposure to uterine factors such as estradiol surge during the morula to blastocyst transition, *Wnt-11* demonstrates upregulation during *in vitro* blastocyst development (78). In addition, the expression patterns of Wnt ligands differ throughout the blastocyst, with *Wnt-1* predominantly expressed in ICM, *Wnt-3a, -6, -7b* and -*10b* in the whole blastocyst, and *Wnt-9a* in mural trophoblast and cells that surround the forming blastocoel cavity, demonstrated with whole-mount *in situ* hybridization (81, 82). Single-cell RNA-seq profiling of late human blastocysts demonstrate upregulation of the genes encoding the Wnt signaling pathway receptors in epiblast cells (EPI) and primary outgrowth during human embryonic stem cell derivation (83).

Maternal/zygotic *Ctnnb1* null blastocysts undergo normal first and second lineage specification. However, the blastocysts are small and undergo fission after hatching or removing the zona pellucida. Fission results in the formation of trophoblastic vesicles that can undergo decidual reactions but not sustain further embryogenesis. Therefore, Messerschmidt et al. suggest that β-catenin-mediated adhesion is important to maintain adhesion between blastomeres at the compaction and blastocyst stage to enable subsequent embryonic development and prevent fragmentation (84).

Wnt signaling is necessary for the allocation of ICM and TE lineages in the blastocyst, regulating hatching, and ensuring blastocyst competency for implantation (21, 85–87). Several studies found that activation of Wnt signaling was inversely correlated with the ability of blastocyst hatching. For example, pig blastocysts treated with Dkk1 demonstrate an increased ability to hatching on day 7 and day 8 of pregnancy (85). Similarly, in mouse blastocysts, Wnt activation by LiCl significantly decreased blastocyst hatching rate in a dose-dependent manner, in addition to decreased subsequent adhesion and outgrowth on fibronectin (22). In bovine blastocysts, activation of the canonical Wnt pathway by Wnt agonist AMBMP caused reduced development to the blastocyst stage (88). In contrast, in a different study, exogenous activation of canonical Wnt pathway *via* 6-Bio treatment enhanced bovine blastocyst development and hatching rate, with significantly increased *Oct4* in ICM and decreased *Cdx2* expression within the ICM and TE compared to the control (89). This suggests that the Wnt pathway positively affects the maintenance of pluripotency marker genes synergistically with PPARδ expression during first lineage diversification (89, 90). These results are in line with previous reports in pig blastocysts where Wnt activation through LiCl treatment causes a lower TE/ICM ratio due to a reduced number of total blastomeres and TE cells (85), in bovine embryos where Wnt inhibition by Dkk1 exposure during morula to blastocyst transition causes increased differentiation into TE and hypoblast lineage (86), and in ICM-derived embryonic stem cells where Wnt activation provides maintenance of pluripotent state and undifferentiated phenotype (91–93). In another study, the inactivation of nuclear β-catenin signaling in mouse blastocysts did not affect the development to the blastocyst stage. However, blockage of nuclear β-catenin accumulation significantly reduced *Cdx2* expression in TE and impaired normal implantation, potentially by downregulating RhoA GTPase, causing disassembly of AJs and cytoskeletal reorganization (21). Likewise, in human embryos treated with β-catenin degrading drug Cardamonin, blastocysts development rate, *CDH1, Nanog, and Sox2* expression levels were unaffected. However, significantly fewer TE cells with *Cdx2* positivity supported β-catenins’ role in TE lineage specification (23). The downregulation of β-catenin not interfering with development until the blastocyst stage can possibly be explained by the ability of plakoglobin to rescue the function of β-catenin (23, 94).

With exogenous Wnt stimulation, there is enhanced spatial overlapping of PPARδ and β-catenin under immunofluorescence analysis. Similar to the effects of canonical Wnt/β-catenin inhibition, blocking of PPARδ in bovine blastocysts causes significantly reduced cell proliferation ratio, blastocyst quality, cell invading ability, and weak blastocyst attachment (89). For instance, overexpression of Dkk1 blocks the activation of dormant blastocysts for implantation in response to E_2_ injection on day 7. However, exposure to Wnt-3a partially overrides this effect *via* nuclear β-catenin stabilization and PPARδ expression as 11% of Wnt-3a treated blastocysts gain implantation competency, highlighting the necessity of nuclear β-catenin signaling in blastocyst activation for implantation (21). In addition to the synergistic effects of PPARδ and β-catenin on proliferation and lineage specification, the coordination of Wnt-β-catenin with PPARδ signaling regulates lipid metabolism, enhancing the blastocyst development and implantation potential (21, 89, 90). Lipid metabolism and ATP generated through fatty acid oxidation is an important energy source for early embryonic development (95, 96). When Wnt stimulation induces PPARδ activity, there is a significant reduction in lipid droplet content, indicative of a high fatty acid oxidation metabolism (89).

Furthermore, Wnt signaling is indispensable for early placentation, trophoblast invasion and differentiation (97). Wnt-3a induces cytoplasmic accumulation of active β-catenin and translocation into the nuclei of differentiating trophoblast stem cells causing upregulation of c-Myc and PPARδ, establishing Wnt-β-catenin signaling as a regulator of trophoblast differentiation (21, 90). High level of β-catenin accumulation in the TE cells is correlated with an increase in cell migration and invasion capacity (90). Similarly, in human embryos exposed to Wnt-3, trophoblast specific marker *EOMES* is upregulated, supporting the necessity of Wnt signaling for TE specification to the trophoblast lineage (23). More studies with genetic manipulations of PPARδ will help enlighten the coordination between Wnt and PPARδ during early embryonic development.

Haegel et al. showed that embryonic ectoderm and mesoderm development is significantly disrupted in β-catenin null mutant mouse embryos (75). In mouse embryos derived from oocytes expressing a stabilized form of β-catenin resistant to GSK3β-mediated proteosomal degradation, development to the blastocyst stage is morphologically normal. However, mutant embryos exhibit a distinct phenotype at E6.5, with a less expanded and disorganized embryonic portion. Cells of the embryonic ectoderm in early postimplantation embryos change their fate, leading to premature epithelial-mesenchymal transition and loss of E-cadherin transcription, supporting the necessity of β-catenin in developing tissues derived from ICM and the maintenance of differentiation potential (98). Therefore, Wnt-β-catenin signaling also contributes to proximo-distal patterning, possibly by generating a symmetry-breaking signal and distinct cell-specification in ICM (99).

## 3 Desmosomes

Desmosomes are adhesive intercellular junctions that tether intermediate filaments (IF) and provide mechanical stability and tissue integrity against mechanical stress (100). Although AJs and desmosomes both confer adhesive properties to tissues, three factors set desmosomes apart from AJs: IF’s ability to stretch to multiple times its original length, IF’s capacity to withstand higher tensile loads than actin and desmosomes’ potential to transform into calcium-independent “hyper-adhesive state” (101). Ultrastructurally, desmosomes are disc shape electron-dense plaques which are 0.2-0.5 μm in diameter. They are composed of a 20 nm thick dense outer plaque, a 7 nm thick dense inner plaque where IF makes a loop attachment and a 34 nm intercellular space with a discrete electron-dense midline creating a mirror image arrangement at cell contact sites (102). Prominent midlines are a characteristic of hyper-adhesive desmosomes, whereas Ca-dependent desmosomes exhibit a somewhat amorphous intercellular space (103).

Desmosomes comprise a membrane core of desmosomal cadherins, desmogleins and desmocollins; a cytoplasmic plaque of armadillo proteins, plakoglobin and plakophilins; and a cytoskeletal adaptor that mediates IF anchorage, desmoplakin (101, 102, 104) (**Figure 1**). Desmosomal cadherins, which constitute desmogleins and democollins, are transmembrane glycoproteins that comprise four extracellular cadherin homology domains, each separated by calcium-binding motifs, a fifth extracellular anchor domain, a single transmembrane domain and a cytoplasmic domain consisting of intracellular anchor and a cadherin-like sequence (ICS). Desmogleins additionally pose unique motifs like a proline-rich linker domain (IPL), a repeat unit domain (RUD) and a desmoglein terminal domain (DTD) (104). There are three isoforms of desmocollin (Dsc1-3) and four isoforms of desmoglein (Dsg1-4) in humans (102). Each of these three isoforms of desmocollins can be alternatively spliced to generate longer “a” and shorter “b” forms that differ in their ICS domain length. Plakoglobins (PK), β-catenin orthologue, and plakophillins (PKP), member of the p120-catenin subfamily, act as a bridge between the ICS domain of desmosomal cadherins and desmoplakin through their central armadillo domain. Structurally, in addition to amino- and carboxyl-terminal domains, PK contains 12 arm repeats, and PKP contains 9 arm repeats with an insert between the fifth and sixth repeats that creates a bend in its morphology (104). Plakin family member desmoplakin, the adapter that couples IF to the desmosomal plaque, contains globular amino and carboxyl terminals and a central a-helical coiled-coil rod domain. Glycine-serine-arginine rich domain (GSR) of desmoplakin carboxyl-terminal mediates the binding of IF (102, 104).

Desmosomes first assemble at cell-cell contact points of TE in the early blastocyst (32-cell stage), in close relation to the onset of blastocyst cavitation and increase in number as the blastocoel expands (105, 106). Immunoprecipitation studies show that desmoplakins and desmogleins are absent in unfertilized eggs. The earliest detectable desmosomal constituent is plakoglobin at compaction (72 h post hCG injection) followed by desmoplakin 1 and 2 at the 16-cell stage (84 h post hCG). Finally, desmogleins and desmocollins start to be detected at the early blastocysts (96-100h post hCG) (105).

Immunocytochemistry studies reveal that desmoplakin (DP) 1&2 first appears as faint punctuate stains along with lateral membrane contact sites after division into 32-cell stage, 2-4h hours post-cavitation, localized exclusively to outer polar TE cells. DP staining becomes stronger in later blastocysts, 12-48h post cavitation. It may be argued that as DP expression in the TE is concomitant with blastocoel cavity formation, DP might be essential in mechanical stability against stress during blastocoel cavity formation (105). However, homozygous DP^(-/-)^ mutants form a TE layer, blastocoel cavity and proceed through implantation (107). Nevertheless, during post-implantation, embryos die by E6.0-E6.5 because of defects in extra-embryonic tissues due to disruption of the keratin network and a dramatic reduction in the number of desmosomal-like junctions at endoderm and ectoplacental cone (106, 107). When Dp^(-/-)^ embryos are rescued by supplementing with Dp^(+/+)^ extra-embryonic tissues, they die shortly after gastrulation, approximately at E12.5, due to overall heart architecture defects, malformed neuroepithelium, collapsed neural tube, fragile skin epithelium and significantly reduced and disrupted capillaries (106). Therefore, DP is essential in early embryonic development through anchoring and maintaining keratin intermediate filament network and assembling/stabilizing desmosomes, all of which are at the very least critical during egg cylinder formation and development of surface ectoderm that can withstand mechanical stress enabling post-implantation growth (107).

Plakoglobin (PG) mRNA is detected very faintly at unfertilized eggs and 2-cell stage embryos and increases from 8-cell stage onwards. Immunoblot studies demonstrate very low abundant PG protein levels from unfertilized egg until early morula stage, with an increase from late morula satge onwards (14). PG are present in the majority of late uncavitated morula under the immunofluorescence microscope as faint linear staining at borders between outer cells. In cells clusters derived from 2/16 couplets, PG linear membrane localization exhibits a similar pattern to DP 1&2, occurring after division into a 32-cell stage (105). However, while 2/16 cell cluster studies reveal that DP 1&2 staining only occurs at presumptive TE cells of cavitated embryos filled with fluid, PG is detected both in cavitated and non-cavitated cell clusters indicating its independence from blastocoel formation. PG also demonstrates a different spatial regulation than DPs. At the onset of PG labeling in cell clusters, PG appears in all cell-cell contact points, including ICM, in probably a non-desmosomal localization associated with vinculin or α-actinin. Later on, PG becomes localized to TE in late blastocysts, like other desmosomal proteins (105). Although the inactivation of *plakoglobin* gene does not affect basic morphogenetic events until early post-implantation, PG mutant embryos die from E10.5 onwards due to severe heart defects, especially in ventricular and atrial trabeculae and endocardial cushions (108).

Desmogleins (DSG) are first detectable as faint punctate stains under immunofluorescence microscopy 2-4 h post-cavitation in early blastocysts and become prominent in TE lateral membranes of expanded blastocysts 12-48 hours post-cavitation (105). Germline inactivation experiments of DSG2 indicate that DSG2 function is essential for embryonic viability at the time of implantation. All DSG2^(-/-)^ mice and a considerable number of DSG2^(+/-)^ mice show a lack of decidual reaction and die at or shortly after implantation (109). Loss of DSG2 also affects the distribution of DP, disturbing its normal localization at cell borders of the blastocyst (109).

Desmocollin 2 mRNA (DSC2) is detected in cumulus cells, unfertilized eggs, 2-, 4-, pre-compact and 16-cell stages with the exclusion of compact 8-cell stage, coinciding with maternal DNA degradation, indicating the contribution of both maternal and embryonic genomes in desmocollin expression (110). DSC3 mRNA is present in unfertilized eggs as well as pre-compaction 8-cell stage embryos (111). Both a and b isoforms of DSC2 and DSC3 are transcribed in the pre-implantation embryo (110, 111). *DSC2* transcription through the embryonic genome is initiated at 16-cell and 32-cell stages just before DSC protein starts to become detectable by immunoprecipitation (110). The earliest detectable linear expression of DSC2 and 3 is present in expanding blastocysts, 12-48 h post cavitation, confined to TE cells and becomes punctate in late blastocysts. So, the maturation pattern of desmosomal proteins under immunofluorescence labelling appears as a change from linear to punctate distribution, restricted to TE cells, with exclusion of PK (105, 111). However, DSC3 might not be restricted to classical desmosomes of early developmental stage embryos. Den et al. showed a similar staining pattern for DSC3 to E-cadherin and β-catenin: linear staining along cell-cell borders of TE cells. This staining pattern may indicate DSC3’s role in maintaining cell-cell adhesion and mechanical integrity of early cleavage-stage embryos alongside E-cadherin. DSC3 staining is also observable in the cytoplasm of oocytes with immunohistochemical staining of ovary sections, indicating that DSC3 is not specifically distributed to desmosome-like cell junctions (111). Dsc3^(-/-)^ homozygous mutant embryos die before compaction is completed, most disintegrating within E2.5 (111).

There is a close relationship between cavitation and desmosome biogenesis, so cavitation might be a prerequisite for triggering desmosomal cadherin synthesis. Correspondingly, desmosomes may be essential in stabilizing TE against fluid pressure within the blastocoel during blastocyst expansion (105). As DSG and DSC expression supersedes the synthesis of desmosomal plaque proteins and coincides with desmosome assembly in TE during cavitation, Collins et al. proposed that DSC2 expression regulates desmosome biogenesis (110).

Intermediate-sized (7-11 nm) filaments produced during pre-implantation embryo development are bundles of cytokeratin filaments and are present in the outer cells of morula and the trophectoderm in close association with desmosomal structures (112). Cytokeratin filament polymerization requires one acidic cytokeratin, ENDO B, and one neutral/basic cytokeratin, ENDO A (113). Synthesis of ENDO A and ENDO B is detected in low levels at the 4-to-8 cell stage by immunoprecipitation (114). mRNA of *ENDO* A was detected in 8-cell embryos; however, 4-cell embryos were not probed directly (115). Cytokeratin filaments initiate to assemble in a cell-autonomous manner influenced by differential contact patterns. There is an extensive filament network formation in the outer cells compared to relatively low levels in the inside cells (113). In the late morula stage embryo, intermediate-sized filaments are related to small areas of nascent desmosomes. In the blastocyst, tonofilament bundles are anchored to normal-sized desmosomes with typical architecture. Just like desmosome-like junctions, intermediate filament structures are not found in ICM (112).

*De novo* keratin network biogenesis has been demonstrated in homozygous keratin 8-YFP knock in mice, that produces fluorescence-tagged keratin 8. 6-8 hours after compaction, diffuse cytoplasmic signals appear and increase continuously at cell borders with maturation into the blastocyst stage, perfectly co-localizing with DP and immediately next to DSG2 (116, 117). The punctate accumulation in TE of the early blastocyst evolves to a pearl-on-a-string model consisting of elongated puncta connected by subcortical filaments in the late blastocysts. Moch et al. suggest that nascent desmosomes serve as nucleation sites for elongating keratin filaments. With subsequent desmosomal fusion, keratin filament anchorage, elongation and bundling, the keratin-desmosome connectivity is stabilized (117).

Desmosomes of the trophectoderm become hyper-adhesive in the blastocyst stage, proceeding from E3 to E4.5. In most early blastocysts, DP is internalized under low-calcium medium incubation (118). However, late blastocysts show DP staining on their plasma membranes, with no sign of DP internalization under low-calcium medium incubation, indicating the desmosome’s transformation into a Ca-independent state. The acquisition of hyper-adhesiveness is correlated with junctional maturation. While Ca-dependent desmosomes of early blastocysts are ultrastructurally rudimentary, without a distinct midline, little IF attached to poorly developed cytoplasmic plaques; Ca-independent hyper-adhesive desmosomes of late blastocysts are mature junctions with well-developed cytoplasmic plaques and abundant IF and a prominent electron-dense midline (118).

Desmosomal re-arrangement between uterus and TE is one of the various factors contributing to successful implantation into the endometrium. In murine maternal luminal epithelium, desmosomal adhesions between trophectoderm cells are downregulated and re-arranged between uterus and trophectoderm, facilitating endometrial invasion (119). Desmoplakin protein expression progressively decreases during the pre-implantation period of uterinal epithelium and becomes barely detectable by days 4.5-5 of pregnancy (120). After hatching, initially hyper-adhesive desmosomes need to weaken during migration of trophoblast cells and revert to calcium dependence. When desmosomal adhesiveness was evaluated in E4.5 blastocysts for 96 h in a low calcium medium, during the time which extensive migration of the TE occurs, internalization of DP molecules was demonstrated. In contrast, blastocysts in a standard medium demonstrated desmosomal junctions in cell-cell contact sites. These changes under a low calcium medium indicate a migratory phenotype where desmosomes are reverted to a calcium-dependent state to enable trophectoderm invasion and successful implantation of the embryo (118).

## 4 Tight junctions

TJs, also named zonulae occludens, are the most apical structures of the apical junctional complex, followed by AJs and desmosomes (121). They seal the paracellular space creating a near leak-proof permeability barrier. TJ have two main functions: fence and gate function (5). As the fence function, TJ provides membrane polarity by separating the plasma membrane into apical and basolateral domains, and creating an asymmetry regarding the composition of cytosol and plasma membrane proteins and lipids. As the gate function, TJ establishes a paracellular diffusion barrier between sealed cells and regulates the passage of solutes and ion selectivity. The structure of TJ greatly affects the transepithelial junctional resistance, thus the transepithelial permeability characteristics (122). TJ barrier function contributes to the structural integrity of cellular sheets and the prevention of invasion of pathogens. Moreover, TJ regulates cytoskeletal protein organization, controlling the actomyosin contractility and distributing cytoskeletal generated tensional forces (6).

TJ comprises three main transmembrane proteins: junctional adhesion molecules (JAM), claudin and occludin. Scaffolding proteins such as zonula occludens proteins (ZO-1, ZO-2, ZO-3) and cingulin associate occludin, claudin and JAM in tight junctional strands, promoting polymerization. These cytosolic plaque proteins also integrate inside and outside signaling and enable binding with cytoskeletal-associated proteins (123) (**Figure 1**).

During embryonic development, TJs have critical roles in several morphogenetic events: 1) Generating apicobasal polarity within blastomeres. 2) Mediating differential AMOT localization at apical membranes of outer cells affecting the cell fate decisions. 3) Creating a functional barrier that triggers cavitation and formation of a fluid-filled blastocoel through hydraulic fracturing and basolateral Na^+^/K^+^ ATPase mediated fluid expansion. 4) Maintaining blastocyst integrity through paracellular sealing (65, 67, 124–125). Apicobasal polarity established in earlier stages of development has two important roles subsequently during blastocoel cavity formation in the blastocyst stage: 1) generation of transepithelial transport by basolateral Na^+^/K^+^ ATPase *via* establishment of an electrochemical gradient and osmotic flow of water and 2) apical TJ biogenesis forming a permeability seal that prevents blastocoel collapse (67, 126). Role of individual TJ proteins regarding these specific events throughout the embryo development will be explained separately.

### 4.1 Claudins

Claudin family is composed of four transmembrane domains, two extracellular loops, a short cytoplasmic turn, and an amino- and carboxyl-terminal containing a PDZ binding motif, which binds to PDZ domain-containing plaque proteins such as ZO (127). Claudin is the major structural component of TJs, forming the backbone of TJ strands, whereas other proteins regulate TJ dynamics (128). It is the primary regulator in defining TJ functional properties, such as creating a tight paracellular cleft and regulating paracellular permeation. Therefore, the claudin family members are subdivided into sealing and channel-forming proteins (6). It has been shown that overexpression of claudin-1 in MDCK cells causes decreased paracellular flux and increased transepithelial electrical resistance, supporting its role in epithelial barrier function (129). Claudin’s sealing role is supported in various claudin knockout experiments, as Cldn1^(-/-)^ mice die within a day after birth as a result of excessive dehydration, Cldn5^(-/-)^ mice show a severely disturbed blood-brain-barrier and die within a day of birth and Cldn 11^(-/-)^ male mice show infertility due to disruption of blood-testis barrier and spermatogenesis (130). Claudin can associate with enzymes such as protein kinases and matrix metalloproteinases (38) and are post-translationally regulated on their CxxC motifs by palmitoylation and phosphorylation (127).

Out of 24 members of the claudin gene family, only *Cldn 4*, *6*, *7* and *12* are detected with RT-PCR starting from the pre-compact 8-cell stage of the mouse embryo. In double immunofluorescence staining with occludin; claudin 4, 6 and 7 were detected as punctate stains in some of the compact 8-cell stage embryos in cell-cell contact regions and converted into continuous bands in every 16-cell stage embryo. In blastocyst, claudin and occludin are co-localized at the most apical regions of the cell interface in TE. To evaluate the contribution of claudins to TJ seal and blastocyst formation, C- terminal of Clostridium perfingens enterotoxin (C-CPE) was introduced into culture medium to bind with extracellular domains of claudin 4 and 6. The barrier function of C-CPE treated mouse embryos was significantly disrupted, interpreted by infiltration of FITC-dextran, a 4kDa permeability tracer, into the blastocoel cavity at E4.0 (131). Ordinarily, the permeability seal should have been established around E3.5. Morphologically, blastocoel cavitation was mostly immature, and in those with a definite blastocoel, expansion was inhibited. Furthermore, C-CPE treatment caused a developmental delay of the mouse embryos, as Oct 3/4, a pluripotency marker, was expressed in all cells at E4.0, which should have been excluded to ICM by E4.0. However, final differentiation was not affected as Cdx2 was expressed in TE and Oct 3/4 at ICM, thus establishing cell fate (131). Similar effects were seen in the knockdown of *Cldn7* in porcine embryos. *Cldn7* knockdown reduced the expression of genes related to TJs *(Ocln, Cldn4, Cldn6, Cdh1, Tjp1, Tjp2*) and several others associated with cell polarity, cytoskeleton and H_2_O channels. Consequently, TJ paracellular sealing was disturbed, as higher FITC-dextran permeability was shown in *Cldn7* knockdown embryos. Furthermore, developmental competence was reduced, but no influence on Sox2 or Cdx2 expression was detected (132).

In single-cell RNA-Seq profiling of human preimplantation embryos, the differential expression pattern of *Cldn10* and *Cldn3* is demonstrated between EPI and TE cell lineages of the cells in late blastocysts, providing insight for understanding gene regulatory networks of embryonic development (33). Similarly, transcriptome analysis of human TE cells demonstrated 2,196 transcripts specific to TE molecular signature, including genes related to junctional proteins such as *Ocln, Dsc2, Dsp, Jup, Pkp4, Gja5*, and *Vcl*. Amongst, *Cldn4* was one of the 100 genes with highest fold change and significant statistical value in TE samples (133). Hernandez-Vargas et al. identified 24 common potential biomarkers related to reproductive outcomes by examining all available endometrial and embryonic omic studies, where *Cldn4* is present for its role in the interaction between the embryo and uterus demonstrated with interactome analysis. Through functional enrichment analysis of embryonic and endometrial interaction networks, ZO-1, occludin and claudin 4 were found to be involved in one of the largest interaction networks at the time of implantation. Therefore, it is important to emphasize the place of claudin within embryonic and endometrial molecular profiles and its contribution to successful preimplantation development and implantation (134, 135).

### 4.2 Junctional adhesion molecules (JAMs)

The IgG-like family of JAMs is composed of single-pass membrane protein, forming homophilic and heterophilic interactions with intrafamily and extrafamily partners and constitute two immunoglobulin-like domains, one transmembrane domain, one cytoplasmic tail containing a PDZ domain, and a zonula occludens protein-binding motif (136). This family consists of three classical (JAM-A, JAM-B, JAM-C) and four related proteins (JAM-4, JAM-L, CAR, ESAM) (136). JAM-A or JAM-1, the family prototype, has a critical role in maintaining cellular polarity as it interacts with the polarity complex protein PAR-3 through its carboxyl-terminal PDZ-binding domain with the Phe-Leu-Val motif. JAM-A also interacts with ZO-1 and afadin, a PDZ domain-containing protein associated with TJ and AJs (38).

During embryonic development, JAM-A is present in embryonic vasculature, inner ear, developing epithelia of lungs, kidneys, skin, hair follicles, choroid plexus and gut tubes (137).

At the mRNA level, *JAM-A* detection by RT-PCR using poly(A) RNA starts at the 2-cell stage, amplifying from the 8-cell stage onwards to the late blastocyst. However, *JAMA1* mRNA starts to be detected in unfertilized eggs when total RNA is used as starting material for RT-PCR. This polyadenylation coincides with zygotic genome activation, compatible with the fact that polyadenylation affects mRNA stability and translational efficiency during the maternal-to-zygotic transition (138, 140). In the human embryo, *JAM* mRNA is ubiquitously expressed throughout development from 3-8-cell stage to the blastocyst (141). In the mouse embryo, JAM-A staining is not detectable in unfertilized eggs, 2-cell and 4-cell stages by immunofluorescence. JAM-A membrane assembly first occurs in the pre-compact early 8-cell stage mouse embryo. While JAM-A localizes in cell-cell contact sites in pre-compaction, its intensity increases in apical microvillus poles with compaction, 6 h after division. At the 16-cell and blastocyst stage, JAM-A re-localizes to cell-cell contacts not only in TE but also in ICM, demonstrating a similar spatial regulation to E-cadherin. Although JAM-A is evident in contact-free basal surfaces of TE cells, it is mainly concentrated at apicolateral regions (138).

JAM has three proposed functions in the preimplantation mouse embryo: 1) maturation of epithelial polarity and cell lineage diversification, 2) early epithelial differentiation and structural maturation of AJs, 3) TJ biogenesis and sealing. Firsly, JAM acts as an anchor for PAR3 binding through its PDZ domain, contributing to the establishment of polarity(142, 143). Concomitantly, PKC ζ is found in the apical membrane at compaction later co-localizing with ZO-1α+ at cell contact sites in the TE, coinciding with the distribution pattern of JAM-1 (138, 144). In Xenophus embryos, the distribution pattern of aPKC has been associated with asymmetric divisions. aPKC is enriched in membranes of fertilized eggs designating the future apical domain, later localizing at apical membranes of 4-cell embryos. After a perpendicular division, membrane-localized aPKC is only inherited by superficial cells, giving blastomeres different developmental potentials due to unequal inheritance (145). Although the role of differential distribution of PKC ζ was not demonstrated in the mouse embryo for cell lineage diversification, Thomas et al. interpreted that JAM association with apical microvilli pole might act as a memory for lineage specification through cytoskeletal organization and cell polarity (138, 146). Secondly, as JAM is unusually assembled on membrane before compaction and all other TJ proteins, it might be essential in early epithelial characterization. JAM-A has been shown to interact with nectin through afadin in epithelial cells. Over-expression of nectin-1 in MDCK cells demonstrated an increased velocity in claudin-based TJ formation as well as E-cadherin based AJ formation (147). Nectin-2-E-cadherin-Jam-1 correlation has also been demonstrated in the mouse embryo, especially in apicolateral contact sites of the morula. However, JAM-A neutralizing antibody showed no effect on compaction of mouse embryo, indicating the independence of compaction initiation and maintenance from JAM-A (138). Thirdly, JAM has a possible role in regulating TJ formation. Overexpression of a mutated form of JAM lacking the extracellular domain disrupts normal localization of PAR-3, aPKC and ZO-1 in MDCK II cells (143). In the mouse embryo, JAM-A neutralizing antibody causes a significant reduction in the cavitation rate of embryos as JAM-A contributes to permeability barrier formation by enhancing intercellular adhesions. (138). Similarly, PKCδ and ζ inhibition significantly delay cavitation (144). However, Anti-JAM-A incubation of early blastocysts do not cause significant FITC-dextran penetration into the blastocoel cavity compared to the control group, indicating the presence of effective TJs (129). Additionally, downregulation of Par3 by RNAi or expression of a dominant negative form of aPKC in 4-cell stage blastomeres bias cells towards becoming ICM. However, it does not affect the appropriate stage-dependent recruitment of TJ components in outer cells (148). 

CXADR/CAR (Coxsackie and adenovirus receptor) functions as a transmembrane component of epithelial TJs and mediates viral recognition of coxsackie B and adenovirus. It comprises two immunoglobulin-like extracellular domains, a single transmembrane domain and a cytoplasmic domain with type I PDZ binding motif that enables interaction with ZO-1, MUPP-1, MAGI-1 and LNX2. N-terminal Ig-like domain 1 homodimerization mediates the cellular adhesion (149). In mouse embryos, several splice variants of transmembrane *CXADR* mRNA *(CXADR 1,2 and 3*) are present. *CXADR1, 2* and *3* are expressed from the 2-cell, morula, and blastocysts stage onward, respectively and demonstrate different subcellular localizations. Protein localization of CXADR starts in the contacts between blastomeres and the cytoplasm at the 4-cells stage. Cytoplasmic localization decreases with compaction, whereas membrane localization enhances to support polarization. In TE cells, CXDR is present primarily at apicolateral membranes, co-localizing with ZO-1, but is also present in ZO-1 free lateral contacts, mediating adhesion (150, 151). In porcine embryos*, CXADR* is detected in all preimplantation stages, with a slight reduction at the 2-cell stage and a sharp increase from the 8-cell stage onwards, by qRT-PCR. CXADR protein is detected within the nuclei, perinuclear region and the cytoplasm at early cleavage stages. With compaction, it becomes localized to the cell membrane, concentrating on the apical side. In the morula and blastocyst stage, CXADR is detected as continuous lines at the apical edge, co-localizing with ZO-1 and occludin. In expanded blastocysts, CXADR accumulates in the nuclei, co-localizing with OCT4. Preimplantation human embryos show a similar distribution pattern for the CXADR protein, localizing in the cytoplasm in early stages and membranous distribution following compaction. In TE of blastocysts, CXADR co-localizes with ZO-1 and occludin in TJs, whereas in ICM, cytoplasmic and nuclear CXADR is prominent. Membrane localization of CXADR is present in ICM facing the blastocoel, precursor of primitive endodermal cells (152).

In expanded and hatched human blastocysts (by normal or assisted hatching process), a switch in *CXADR* RNA expression occurs to prepare TE for implantation by loosening the intercellular connections. Full-length *CXADR* is switched to its soluble spliced variant *CXADR3/7* in expanded, *CXADR4/7* and *CXADR2/7* in hatched blastocysts. *CXADR2/7, CXADR3/7* and *CXADR4/7* do not contain a transmembrane domain, thus accumulate in the cytoplasm. Likewise, the CXADR protein concentrates in the nuclei of TE cells in hatched blastocysts (153).

CXADR knockdown porcine embryos either fail to develop to the blastocyst stage or do not expand fully. Adhesion and TJ function are significantly disturbed, indicated by decreased *CDH1, OCLDN, TJP1, TFAP2C* transcription, disrupted protein localization of E-cadherin, occludin, ZO-1 and a significant increase in FITC-dextran permeation. No effect is seen on transcription levels of *OCT4* and *SOX2* (152). Porcine embryos treated with ROCK inhibitor demonstrate a decreased rate of development to blastocyst stage, lower levels of CXADR, TJP1, OCLN, CDH1 transcription, disrupted localization of CXADR, ZO-1 and impaired paracellular permeability (154, 155). Similarly, mouse embryos treated with CXADR blocking antibody or CXADR siRNA demonstrate higher FITC-dextran permeability into the blastocoel (150, 151). 8-cell stage mouse embryos treated with CXADR blocking antibody that has undergone Ca^+2^ switch demonstrate impaired blastocyst formation with significantly smaller blastocoels and significant downregulation in H19 and Cdx2, indicators of trophoblast lineage commitment (150, 156, 157). In parallel, CXADR siRNA injection into 1-cell mouse zygotes causes reduced blastocyst formation and gene expression involved with AJ *(CDH1*), TJ (*OCLN, TJP1, CLDN4*) and lineage specification (*Nanog, Cdx2, Oct4, TEAD4, YAP1*)(151, 158). In addition, when CXADR knockdown blastocysts are transferred into surrogates, the implantation rate is significantly lower than the control. Half of the knockdown embryos fail to maintain pregnancy after the second trimester, evaluated by gradual weight gain after embryo transfer (158). Therefore, CXDR is involved in cellular adhesion between blastomeres, TJ assembly, TE lineage diversification and pregnancy maintenance during embryogenesis.

Kwon et al. suggest that ADAM10 is involved in regulating TJ biogenesis by providing docking sites for SH3 domains of CXADR and TJP1/ZO-1 with its SH2 binding motif on the cytoplasmic domain. Mouse and porcine embryos treated with ADAM10-specific inhibitor or knockdown for ADAM10 demonstrate a significant decrease in the blastocyst development rate, disrupted AJ/TJ gene expression, defective permeability seal and decreased total area of outgrowth in outgrowth assay (151, 159) (**Table 1**).

**Table 1:** Determinants of junction biogenesis during preimplantation embryo development.

Regulation of Junction Biogenesis		
**Cell-cell Contact**	Culturing 2/8 pairs, derived from the division of isolated 1/4 blastomeres, in Ca+2 free medium and ECCD-1 containing medium disrupts cell polarity axis and ZO-1 distribution pattern.	(148)
	E-cadherin -/- embryos show decreased expression of α- and β-catenin together with loss of ZO-1 localization at cell-cell contact sites and disrupted cortical actin filament organization.	(21)
	A low calcium level causes E-cadherin internalization but does not affect TJ stability.	(108)
	In the pre-compact 8-cell stage, blocking of ECCD-1 and E-cadherin or low Ca^+2^ level disrupts the distribution pattern of desmocollin and desmoplakin in TE cells.	(100)
	Embryos cultured in ECCD-1 from the 8-cell stage until the late morula stage shows either negative or weak staining for occludin. Embryos overcome the effect when culture is extended for a further 4h.	(149)
	SPECC1 is detected at apical cell-cell contacts at the blastocyst stage, and knockdown of SPECC1 disrupts paracellular sealing, reducing the rate of blastocyst development.	(150)
	LIMK is essential for early cleavage compaction through AJ assembly and actin filament organization. LIMK1/2 knockdown in porcine embryos causes abnormal cleavage, reduced blastocyst formation, disrupted localization of β-catenin, E-cadherin, ZO-1, CXADR and decreased cortical actin levels.	(151)
	Suppression of p38 MAPK signalling causes reduced blastocyst expansion and cavitation accompanied by increased TJ permeability. Although E-cadherin localization remains normal, ZO-1 displays punctate localization and loss of co-localization with F-actin.	(152)
	Mouse morula treated with ADAM10 inhibitory peptide demonstrate retarded blastocyst development and disrupted localization pattern for CXADR and ZO-1.	(140)
	ADAM10 knockout significantly decreases porcine blastocyst formation rate, transcription of Cxadr, Ocln, Tjp1, Chd1, Cldn6 and increases paracellular permeability (159).	(159)
**Asymmetric contact pattern**	The differential distribution of ZO-1 among polar and nonpolar cells is a consequence of the change of cell contact pattern from symmetric to asymmetric, initiating ZO-1 down-regulation in ICM. Isolated ICM can still regenerate into TE lineage, as mRNA’s of ZO-1 are inherited to nonpolar cells, but symmetrical contact inhibits their translation. With the re-establishment of contact asymmetry, ZO-1 synthesis can start *de novo* at isolated ICM’s newly formed outer cells at the compacted 8-cell stage, slightly after intercellular flattening and cell polarization.	(153)
	In asynchronous cell aggregates of 1/4 + 1/8 cell couplets, the incidence of ZO-1 assembly is reduced by %50 and appear as randomly distributed spots, indicating the necessity for simultaneous competency in companion cells.	(148)
	*DSC2* shows a differential expression pattern between TE and ICM. In isolated ICMs, there is a seven-fold increase in *DSC2* transcription and assembly of desmocollins and desmoplakins at cell-cell contact sites.	(100)
	Isolated ICM demonstrate an identical but accelerated membrane junction assembly pattern of TE lineage.	(154)
**Blastomere Polarization**	Late morula cultured in the presence of PKCδ activator peptide show enhanced membrane PKCδ distribution and increased rate of cavitation in a dose-dependent manner by tightening the TJ seal. In contrast, PKCδ and ζ inhibitory peptides cause delayed cavitation but do not affect membrane assembly of TJ proteins.	(155)
	TJ proteins are differentially regulated by PKC isotypes. In isolated ICM, indolactam- and TPA- mediated PKC activation stimulates membrane assembly of ZO-2 and ZO-1 α +, and only ZO-1 α +, respectively. Both activators increase the membrane pool of PKCδ, while PKC ζ shifts to the membrane only upon TPA activation to co-localize with ZO-1 α +.	(134)
	Isolated ICMs cultured in PKCδ translocation inhibiting peptide and PKC ζ pseudosubstrate inhibitor show significantly reduced ZO-2 and both ZO-1 α + and ZO-2 membrane assembly, respectively.	(154)
	*Pard6b* knockdown embryos fail to form a blastocyst cavity due to defective paracellular sealing and abnormal ZO-1 and actin distribution.	(54)
	ADAM10 knockdown in mouse embryos causes failure of blastocyst development, increased FITC-dextran diffusion, and decreased *Cdh1* and *Pard6b* expression.	(140)
	Downregulation of Par3 by RNAi or expression of a dominant negative form of aPKC in 4-cell stage blastomeres biase cells towards becoming ICM. However, it does not affect the appropriate stage-dependent recruitment of TJ components in outer cells.	(156)
	cPKC inhibition inhibits trophectoderm migration during implantation, which typically requires the nature of desmosomes to switch to a Ca-dependent state.	(108)
	Rab 13, a small GTPase, which is present within the cytoplasm before AJC formation, demonstrates a precise co-localization with ZO-1α - in compact 8-cell stage embryos and remains concentrated within apicolateral contact sites of outer cells of the morula and TJs of TE.	(157)
	Rac 1, a small GTPase, is localized adjacent to cell membranes of 2- and 4- cell stage blastomeres and shifts to the cytoplasm with compaction.	(158)
	Downregulation of β1 subunit by Na/K-ATPase β1 subunit siRNA microinjection disrupts appropriate cortical distribution of ZO-1 and occludin, thus preventing TJ maturation and establishment paracellular permeability barrier.	(113)
	Embryos treated with ouabain, a specific inhibitor of Na/K ATPase, or cultured in a K+-free medium demonstrate discontinuous ZO-1 and occludin distribution, as well as increased FITC-dextran permeation into the blastocoel.	(159)
**Gap Junctional Communication**	Cx43 knockdown causes obliteration of ZO-1 in porcine blastocysts, causing an increase in cell permeability.	(160)
	Chemical inhibition of gap junctional coupling in 8-cell stage embryos and ICM cells, did not affect membrane assembly of E-cadherin, ZO-2, ZO-1 α +, occludin and desmoplakin and specific PKC distribution patterns were still preserved.	(154, 155)
**Cellular metabolism**	AMPK inhibition inhibits preimplantation embryo development by impairing TJ seal and disrupting of ZO-1 protein localization in blastocysts causing collapse.	(161)
	In DDK embryos with natural GJIC compromise, exposure to membrane-permeable cAMP or adenylate cyclase activator increases GJIC and survival to the expanded blastocyst stage.	(162)
	Gap junction permeability is very sensitive to pH, and when the pH is lowered in the medium, the GJs disrupt and compaction fails. The embryo cannot reach the blastocyst stage.	(162)
**Transcription factors**	Knockdown of AP-2 γ (Tcfap2c)), a novel upstream regulator of *Cdx2* expression, causes downregulation of Pard6b, Tjp2, Cldn4, Cldn6, Cldn7 and prevents TJ formation and paracellular sealing at apical cell borders, inhibiting blastocyst formation	(163)
	Cdx2 ^-/-^ embryos fail to maintain blastocoel and epithelial integrity due to disrupted localization of ZO-1 α -, ZO-1 α + and E-cadherin.	(164)
**Actomyosin skeleton**	Porcine embryos treated with ROCK inhibitor demonstrate a decreased rate of development to blastocyst stage, lower levels of *CXADR, TJP1, OCLN, CDH1* transcription, disrupted localization of CXADR, ZO-1 and impaired paracellular permeability.	(141)
	Applying ROCK inhibitor H-1152 to inhibit myosin II activity immediately after the initial contact formation between neighboring actin rings results in uncoupling of the rings and reduced E-cadherin and ZO-1 at junction sites.	(165)
	E4.5 embryos treated with ROCK inhibitor H-1152 to reduce actomyosin contractility, shrink due to reduced cortical tension and display an increased number of cytoplasmic ZO-1 and discontinuous junctional ZO-1 on the plasma membrane.	(166)
	In mouse early embryo, Cytochalasin D mediated disruption of microfilaments prevents intercellular flattening, delays the establishment of cell polarity with respect to the contact point, slows and disrupts assembly of ZO-1.	(148)
**Epigenetic modifications**	CBEP2, an mRNA-binding protein, regulates Tjp1 mRNA subcellular localization and stabilization *via* binding to the cytoplasmic polyadenylation element in the 3’UTR on Tjp1 mRNA. CBEP2 knockdown mouse embryos demonstrate impaired TJ and blastocyst formation and mislocalized Tjp1 mRNA with decreased variation in poly (A) tail length.	(167)
	CBEP2 depletion by RNAi in porcine embryos causes reduced blastocyst development, impaired TJ seal, and disrupted localization of ZO-1, CXADR, occludin, but no significant changes were seen at transcriptional level, in parallel with a posttranscriptional defect involving mRNA stability.	(168)
	Posttranslational modification by protein O-mannosylation is essential for cadherin-based cell adhesions, blastocyst development and embryonic viability. Mouse embryos, where O-mallotransferase is pharmacologically blocked, show disrupted E-cadherin and ZO-1 localization and reduced blastomere attachment.	(169, 170)
	Knockdown of INO80 in porcine embryos impaired blastocyst development, paracellular sealing of TE, and decreased expression of OCT4, CDX2, TEAD4, CDH1, OCLN and several cell polarity, cytoskeleton and fluid-accumulation related genes.	(171)

### 4.3 Occludin

Occludin is an approximately 65 kD four-pass transmembrane protein, consisting of two extracellular loops, an intracellular turn, amino- and carboxyl-terminal, which directly associates with ZO-1, ZO-2, ZO-3 and actin (127). However, it can also use an adapter such as cingulin to interact with actin and myosin through cingulin’s head and central rod domain, respectively. This interaction of occludin-cingulin-actomyosin enables the transduction of mechanical force created by actin-myosin contraction. Occludin’s ability to associate with enzymes aid post-translational modifications during TJ assembly, primarily through aPKC on serine/threonine (38).

Occludin mRNA is detected in cumulus cells, unfertilized eggs and all pre-implantation stages including late blastocysts, showing a decline during the 4-cell stage, presumably indicating maternal mRNA degradation. Isolated ICM of early blastocysts also contain occludin transcripts. Under confocal microscopy, early cleavage, pre-compact and compacted 8-cell stages show weak diffuse cytoplasmic staining and punctate perinuclear staining at the 16-cell stage. Occludin localization at the apicolateral membrane contact sites first occurs in late morula, with precise co-localization with ZO-1α+. In blastocysts, occludin shows a cell-specific localization to TE. Although its transcription is not cell-specific, occludin proteins are not assembled in ICM. (160). With a minor difference, Moriwaki et al. started to detect occludin as punctate concentrations along cell-cell contacts in some of the compact 8-cell stage embryos that evolved to linear staining patterns in most embryos at the 12-cell stage. At the 16-cell stage, occludin was co-localized with claudin at every cell-cell contact site. (131). We believe this difference arises from the epitopes used, as Sheth et al. used rabbit anti-human occludin antibody and anti-chick occludin antibody, whereas Moriwaki et al. used rat anti-mouse occludin monoclonal antibody.

Actomyosin cytoskeleton regulates occludin-dependent control of barrier function through ZO proteins (161, 162). Hence, ZO-1α+ scaffolding to the cytoskeleton is critical for establishing functional occludin anchoring. Immediately after assembly at AJC and just prior to cavitation of the embryo, occludin is converted from Triton X-100 soluble form to insoluble form, through phosphorylation. This switch in Triton X-solubility rate, indicates a distinct role for occludin in TJ assembly in TE and blastocyst formation through TJ sealing. This phosphorylation may also be responsible in regulating occludin association with ZO-1α+ (160). 8-cell, early and late morula stage mouse embryos cultured in presence of occludin antibody demonstrate significantly lower rate of blastocoel formation which have significantly lower mean diameters, volume and increased permeability to FITC-dextran, indicative of a disrupted permeability seal. Culturing embryos with occludin antibody also affects trophoblast differentiation where H19, whose expression correlates with trophoblast differentiation, is not detectable in RT-PCR. Whereas, Oct-4 expression is significantly higher in embryos exposed to antibody compared to the controls (163).

### 4.4 Cingulin

Cingulin, an actomyosin and microtubule-associated scaffold, was first identified in 1998 by immunofluorescence detection of monoclonal antibodies against brush border myosin from intestinal epithelial cells (164). It is a parallel homodimer consisting of a globular head-domain that interacts with actin, microtubules, ZO proteins, a coiled-coil rod domain, and a small globular tail domain. Myosin 2 may associate with cingulin through both head and rod domains. Rho family GTPases modulate cingulin’s association with cytoskeletal elements (165).

Cingulin is detected in unfertilized eggs and all preimplantation stages by western blotting, indicating the involvement of both maternal and zygotic genomes. However, the relative cingulin levels differ regarding embryonic stages, coinciding with the global degradation of maternal mRNA and activation of the zygotic genome. Cingulin synthesis shows a sharp decline in the late 2-cell stage, followed by a low level of synthesis between the late 2-cell and early pre-compact 8-cell stage, 2.3 fold increase at compaction and a linear increase until the late blastocyst stage (166).

Cingulin protein is not only restricted to maturing TJs but is also detectable in the cytocortex and cytoplasm in different stages of development (167). Cytocortical cingulin is detected in the cytoplasmic face of the oolemma and corona radiata cells directly associated with the oocyte. This indicates a possible role for cingulin in cumulus-oocyte interactions, regulating meiosis and oocyte maturation. In the early cleavage stages, cytocortical cingulin is detected on the outer embryo surface. Compacted 8-cell stage embryos show a labile localization of cytocortical cingulin at apical microvillous poles of blastomeres, influenced by cell-cell contacts. Thus, cytocortical cingulin might be involved in organizing cytocortical elements, preferentially myosin (167). Junctional cingulin localization starts as punctuated stains in the early 16-cell stage. It becomes linear in the late 16-cell stage and increases in intensity as blastocysts develop. In the blastocyst, cingulin shows tissue specificity favoring TE cells, with a 15-fold difference from ICM (166). At the 32-cell stage until 12 h after cavitation initiation, transient cytoplasmic cingulin is detectable due to the degradation of cytocortical cingulin (167). In this manner, cytocortical cingulin is not a protein store for TJ biogenesis, considering its labile localization pattern with respect to changes in cell contact and short half-life (167).

Cingulin does not have a role in the initiation of junction assembly. Maternal cingulin is too short-lived to be involved in TJ assembly. Cingulin in unfertilized eggs and 4-cell embryos have 4 h half-life, while the half-life of cingulin is 10 h in early blastocysts. In addition, embryonic cingulin appears to be associated with AJC in 16 cell-stage, unlike ZO-1, which precedes cingulin by 10 h and assembles at nascent junction sites at compaction (166).

### 4.5 Zonula occludens

Zonula occludens (ZO) proteins are members of the MAGUK family and consist of ZO-1, ZO-2 and ZO-3, which are PDZ domain-containing scaffolding proteins that enable the assembly of multiprotein complexes at the cytoplasmic end of junctions (4). While the PDZ-domain associates with occludin (PDZ 2/3) and claudin (PDZ1), COOH-terminal interacts with actin, forming a link to the cytoskeleton (121). This interaction enables cellular adaptation, dynamic regulation of cell shape and movement, cytoplasmic and perijunctional actomyosin organization, contractility regulation and generation of membrane and cortical tension (168). Two nuclear localization signals (NLS) are located at the first PDZ repeat or GK domain, which can target a protein to the nucleus, and a nuclear export signal (NES) sequence that mediates the nuclear-cytoplasmic shuttle in conditions like impaired cell-cell contacts or mechanical injury (169). In addition to its function in scaffolding between cytoplasmic and transmembrane proteins, ZO-1 has a potential role in linking AJs and TJs, though a possible link between occludin and p120-catenin as well as afadin-6. Therefore, ZO-1 might be essential for correct induction of junction biogenesis on the lateral membrane (5). ZO-1 has two isoforms depending on the presence of an internal 80-amino acid a domain (α - and α +). ZO-1 α + is expressed in conventional epithelia, while ZO-1 α – is seen in highly specialized epithelia such as seminiferous tubules and renal glomeruli. This indicates that ZO-1 α – presence is correlated with structurally dynamic junctions, showing a direct relationship with the degree of junctional plasticity (170).

Except for the yolk sac and extraembryonic mesoderm, where only ZO-1 is detected, almost all cell types of the embryo show ZO-1 and ZO-2 co-expression at cell-cell contacts. ZO-3 is co-localized with ZO-1 and ZO-2 only in the outermost epithelial layer (171).

ZO-1 (encoded by *Tjp1*) is essential for embryonic vitality. Tjp1^(-/-)^ mice die at E10.5 due to extraembryonic defects, such as abnormal vasculogenesis in the yolk sac at E8.5, a placenta lacking immature embryonic nucleated erythrocytes and embryonic blood vessels, lack of chorioallantoic fusion at E9.5 and apoptosis defects at notochord, neural tube and hindgut. Grossly, delayed embryonic development and absence of turning is evident by E9.5 in Tjp1^(-/-)^ mice (171). Similar to ZO-1, ZO-2 deficient mice embryos also show morphologically small and abnormal appearance by E7.5 as well as extensive programmed cell death observed by TUNEL assay. This compromises embryo’s viability and causes death shortly after implantation (172). Another study showed that CPEB2, an mRNA-binding protein, regulates Tjp1 mRNA subcellular localization and stabilization via binding to the cytoplasmic polyadenylation element in the 3’-UTR on Tjp1 mRNA. CPEB2 knockdown mouse embryos demonstrate impaired TJ and blastocyst formation and mislocalized Tjp1 mRNA with decreased variation in poly (A) tail length (173). Additionally, CPEB2 depletion by RNAi in porcine embryos causes reduced blastocyst development, impaired TJ seal, and disrupted generation of ZO-1, CXADR, occludin, but no significant changes were seen at transcriptional level, in parallel with a posttranscriptional defect involving mRNA stability (174). Similar results on ZO-1 and ZO-2’s roles in embryo development were also observed in an *in vitro* peri-implantation mouse embryogenesis research model using ZO-1 and ZO-2 gene knockouts in mouse embryonic stem cells. ZO-1^-/-^ZO-2^-/-^ embryoid bodies (EB) show disrupted TJ formation causing disorganization of extraembryonic endoderm, exhibiting discontinuous cell arrangements that lack compactness, presence of sparse microvilli on apical surface and irregular deposition of the basement membrane. Basement membrane is critical for polarization and formation of primitive ectoderm. Respectively, EB showed defected epithelization of primitive ectoderm, eventually leading to apoptosis and creation of excessive irregular cavitations in the interior of EB (175). ZO-3 is dispensable for mouse embryonic development (172). ZO-3^(-/-)^ mice are viable and fertile, with no effect on the morphology of epithelial cells, demonstrating well polarized and characteristic apical membrane specializations and TJ molecular structure *in vivo* (176).

ZO-1 α- mRNA is detected in cumulus cells, unfertilized eggs and in following developmental stages, whereas ZO-1α+ mRNA is not identified in eggs, early/mid cleavage stages and starts to be detected at 44% of 16-cell morula, 89% of late morula and 100% of blastocysts. These transcripts are not tissue specific, detected both in ICM and TE (177). ZO-1 mRNA is inherited by nonpolar cells during differentiative division, supporting the viability of ICM independently from TE-mediated protection. Immunosurgically isolated ICM cells can regenerate TE cells through flattening, envelopment of ICM core by outer ICM cells and translation of TE-specific polypeptides (178). Metabolic labelling and immunoprecipitation of embryos demonstrate that synthesis of ZO-1 α- is detected in low levels until the 4-cell stage (177) and increase with late cleavage. ZO-1 α+ synthesis, on the other hand, is first evident at the blastocyst stage (177). Sheth et al. presented that Rab 13, a small GTPase, which is present within the cytoplasm before AJC formation, demonstrates a precise co-localization with ZO-1 α- in compact 8-cell stage embryos and remains concentrated within apicolateral contact sites of outer cells of the morula and TJs of TE (179). Another study showed that AMPK activation via prolonged AICAR treatment inhibits preimplantation embryo development by impairing TJ seal and disrupting of ZO-1 protein localization in blastocysts causing collapse (180).

In the pre-implantation mouse embryo, ZO-1 membrane assembly occurs in two waves: 1) ZO-1α- localization at cell contact sites of compact-8 cell embryo, facilitated with E-cadherin/catenin complex 2) ZO-1α+ localization at 32-cell stage together with occludin. ZO-1 expression is initially detected as punctate accumulations in cell-cell contacting regions at the compacted 8-cell stage, slightly after intercellular flattening and cell polarization (181) and is strengthened in the following developmental stages, transforming into continuous belt-like structures at the perimeter of each cell in late morula (18, 177, 178, 181). Simultaneously, in late morula, some blastomeres show weak perinuclear staining of ZO-1α+, while in nascent blastocysts, ZO-1α+ is detected in apicolateral contact regions. Although ZO-1 expression does not show cell specificity at the transcriptional level, both ZO-1α- and ZO-1α+ are restricted to TE at the translational level (177, 178). In synchronized cell clusters, ZO-1α+ shows membrane assembly at TJ after division into 32-cell stage and prior to formation of blastocoel cavity (177). Considering the *de novo* transcription of ZO-1α+ at the blastocyst stage, its late assembly in TJs at 32-cell stage just prior to cavitation and ZO-1 α-’s correlation with junction plasticity, Sheth et al. proposed ZO-1α+ as the regulator of the timing of junction sealing (177).

ZO-1α+ and occludin are precisely co-localized in intercellular contacts of blastomeres (160) (177). In epithelial cells, the U5 motif of the SH3-U5-GUK-U6 region of ZO-1 has been critical for occludin binding and TJ assembly (182). As mentioned earlier, Seth et al. demonstrated a similar localization pattern for occludin with ZO-1α+ and suggest that they are processed together, associate in the same intracellular vesicle and ZO-1 α + expression timing regulates the delivery of occludin to the TJ membrane site. They supported their hypothesis by using Brefeldin A, an inhibitor of protein trafficking, which inhibited occludin (160) and ZO-1α+ (177) assembly at TJ sites and blastocoel formation in mouse embryos (170). However, this needs to be reconsidered, as occludin localization to membrane starts to be detected in compact 8-cell embryos by Moriwaki et al.

In porcine embryos, ZO-1 α- is expressed in cumulus cells, oocytes and all preimplantation stages, with a decrease in the expression level between 2-cell and 8-cell stages. ZO-1 α+ is only detected in the cumulus cells, morula and blastocysts stage, with an increase in expression level during the transition from morula to the blastocyst (183).

The actomyosin cytoskeleton and its interaction with TJ proteins is critical in maintaining the developmental competency of the embryo. Linkage to actin enhances the junctional localization of ZO-1 and maintenance of TJ barrier function (161). F-actin binding to ZO-1 induces conformational changes, in which the stretched conformation of ZO is active and enhances scaffolding (184).

In 16-32 cell stages, a cortical F-actin ring assembles in the outer cells of the embryo, expanding to cell-cell junctions. Suppression of p38 MAPK signaling causes reduced blastocyst expansion decreased due to the and cavitation accompanied by increased TJ permeability. Although E-cadherin localization remains normal, ZO-1 displays punctate localization and loss of co-localization with F-actin (185). The rings of neighboring cells form initial contact points, coupling to the same site along the junction. The junctions enable efficient zippering by a localized tension increase due to myosin II accumulation, that is followed by neighboring cells in the opposite side of the initial ring. The zippering triggers junction maturation by recruitment of ZO-1, occludin, E-cadherin, α-catenin and seals the embryo (186). An effective and dynamic seal is essential for blastocoel formation, internal pressurization of the cavity and hatching prior to implantation (187). TJs are the stress-bearing sites during blastocyst expansion. There is a twofold increase in luminal pressure during blastocyst development from E4 to E4.5. This is accompanied by increased cortical tension and cell stretching in TE that leads to occludin and vinculin enrichment at TJ. Matured junctions establish a positive feedback loop that allows the blastocyst to accommodate pressure during growth (125). The embryo hatches after the gradual luminal pressure build-up and volume increase mediated by water transport from aquaporin channels along an osmotic gradient generated by Na^+^/K^+^ ATPase (187). From E3.5 to E4.5, with the increase of inner pressure and tensile forces on TE, cytoplasmic ZO-1 proteins shuttle to plasma membranes to enhance cell-to-cell adhesiveness. Cytoplasmic ZO-1 condensates are regulated in a force-dependent manner. Reducing actomyosin contractility by a ROCK inhibitor, reducing inner hydraulic pressure by inhibiting Na^+^/K^+^ ATPase and mechanically reducing tension by piercing embryos with a glass needle all cause an increase in the number and volume of cytoplasmic ZO-1 punta (188) (**Figure 4**). Embryos treated with ouabain, a specific inhibitor of Na^+^/K^+^ ATPase, or cultured in a K^+^-free medium demonstrate discontinuous ZO-1 and occludin distribution, as well as increased FITC-dextran permeation into the blastocoel (189).

Early 8-cell stage embryos electroporated with ZO-1 siRNAs demonstrate reduced average number of blastomeres in morula and blastocyst formation rate, in a dose-dependent manner. However, ZO-1 disruption does not affect ZO-2 or F-actin distribution, indicating a specific effect of ZO-1 depletion in blastocyst development. In addition, ZO-1 suppressed embryos still undergo compaction and proper membranous E-cadherin localization, demonstrating the independence of AJ formation from ZO-1 (16). Similar results were obtained in different cell lines on the role of ZO-1 in TJ biogenesis. (190, 191). Posttranslational modification by protein O-mannosylation is essential for cadherin-based cell adhesions, blastocyst development and embryonic viability. Mouse embryos, where O-mannosyltransferase is pharmacologically blocked, show disrupted E-cadherin and ZO-1 localization and reduced blastomere attachment (192, 193). When the porcine oocyte-cumulus complex is treated with ZO-1-shRNA1, the blastocysts development rate is significantly reduced, with a lower total cell number and *Nanog* expression. The reduced *Nanog* level indicates lower blastocyst quality and early embryonic developmental capacity (183).

As explained earlier, ZO-1 has a role in differential activation of Hippo signaling pathway and cell fate determination through sequestration of Amot at the apical membrane domain of outer cells (54). Mouse embryos manipulated with ZO-1 siRNA show decreased Cdx2 and Oct-4 expression in the morula stage, indicating ZO-1’s role in differentiation of nonpolar blastomeres to polar trophoblast cells (18).

ZO-2 mRNA is detected in unfertilized eggs and all pre-implantation developmental stages, with a transient reduction at the 2-cell stage due to maternal-to-zygotic transition in mouse. Like other TJ proteins, ZO-2 protein is expressed in a stage-dependent matter. Maternal protein is nuclear associated, with ZO-2 evident in close relation with metaphase spindle at the unfertilized egg. It becomes more abundant within the pronuclei of zygote and nuclei in the 2-cell stage after fertilization. The nuclear localization of ZO-2 remains prominent until compaction and diminishes thereafter (194). ZO-2 shows the first distinct membrane localization at AJC at the late 16-cell stage, co-localizing with E-cadherin. Timing of ZO-2 assembly coincides with cingulin, 12 hours after ZO-1 α- localization, probably through binding sites of ZO-1 or JAM-1. ZO-2 localization is restricted to outer TE cells that demonstrate cell contact asymmetry in late morula and early blastocyst. With the maturation of AJC, ZO-2 segregates from E-cadherin and shows apical co-localization with occludin at the early blastocyst stage, indicating true TJ formation (194). In porcine embryos, ZO-2 is expressed in cumulus cells, oocytes and all preimplantation development stages. Expression levels reach a peak at the 4-cell stage, decrease at the 8-cell stage and increase thereafter (183).

ZO-2^(-/-)^ mouse embryos demonstrate disrupted apical junctional complexes, indicated by the absence of pronounced electron-dense apical junctional plaques under transmission electron microscopy in E6.5 and E7.5. In addition, the permeability barrier is altered, causing increased permeability to lanthanum. However, the symmetric distribution of membrane proteins is not affected (172). In another study using ZO-2 siRNA, ZO-2 depleted mouse embryos show a significantly delayed rate of blastocoel cavity formation, with a 20% reduction in cavitated embryos. However, TJ barrier function, cellular proliferation, and capacity to form outgrowths after hatching from zona pellucida were not affected in ZO-2 knockdown embryos. A compensatory increase in ZO-1 protein by 30% possibly stabilizes the development. This compensatory upregulation in ZO-1 protein level occurs by a post-transcriptional mechanism, as ZO-1 mRNA levels do not change under ZO-2 siRNA treatment (194). The evaluation of the knockdown of ZO-1 alone and with ZO-2 indicated a more prominent role for ZO-1 in TE cell TJ integrity and blastocyst morphogenesis, compared to ZO-2, which has a more supportive role (192).

## 5 Gap junctions

GJs are integral membrane proteins that coordinate the cellular response of heterogenous cells. They function as intercellular channels of communication and allow the transport of small molecules such as amino acids, sugars, intracellular messengers (e.g., cAMP, inositol triphosphate) and ions (e.g., K, Ca, Na) between cells (195, 196). In transmission electron microscope, GJ appear as plasma membrane appositions separated by a 2-3 nm gap (193). The basic structural units of GJ are connexins. Six connexin proteins radially arranged around a pore form a hexamer called connexon. Individual connexons (hemichannels) containing a single connexin type are termed homomeric, while connexons comprising different connexins are termed heteromeric (194). Apposing connexons of adjacent cells create GJs (196) (**Figure 1**). Connexins comprise tetraspan transmembrane domains (TM) with intracellular N- and C- terminus, two extracellular loops between TM1-TM2 and TM3-TM4, and one cytoplasmic loop between TM2-TM3 in the intracellular space (196). While cytoplasmic domains are unique to each connexin with differing length and amino acid compositions, transmembrane regions and extracellular loops may be identical among different connexins (195). Maeda et al. demonstrated the structure of the connexin 26 GJ on channel at 3.5 A resolution. This revealed a pore that narrows from 40 A at the cytoplasmic side of the channel to 14 A near the extracellular side, then widening to 25 A on the extracellular space, giving it an hourglass appearance (197).

Genomic database screenings have detected 20 mouse and 21 human connexin genes. Different GJ proteins are distinguished and named by their predicted molecular mass in kDa (198). Many cells co-express several connexin isoforms. Connexin isoform stoichiometry and different pore diameters determine channel selectivity to biological signaling molecules and metabolites among GJs (199).

Junction-mediated intercellular communication in the form of dye transfer is first detected in early compacted, late 8-cell stage mouse embryos (200–202). Prior to the 8-cell stage, blastomeres may communicate *via* cytoplasmic bridges remaining after cleavage (203).

In human pre-implantation embryos, several connexin isoforms, including Cx26, Cx31, Cx32, Cx43 and Cx45, are detected in transcript and protein levels (204–206). Cx43 is the predominant connexin isoform expressed in the human embryo (205). Cx43 is first detected at 4-cell stage embryos in the perinuclear region and occasionally in opposing cell membranes. In 8-cell stage, Cx43 staining is evident in cell membranes and the cytoplasm. With the development of embryos to the 16-cell stage and an increase in cellular apposition and cell-cell adhesion, the density of GJ labeling increases correspondingly (205). While the GJs of TE are at the outermost edge of cells and are tightly packed, they display an intermittent punctate staining along the plasma membrane of ICM (205). In the blastocysts, Cx31 and Cx45 show complete co-localization with Cx43 as bright punctate stains with immunocytochemistry (204). Embryo evaluation under light microscope is not adequate to evaluate embryo quality. Superficially normal cleavage stage embryos may exhibit nuclear abnormalities with nuclear labeling, which is indicative of poor intercellular communication and Cx43 containing GJs. This can be improved by increasing intracellular pH or cAMP levels and creating optimal culture conditions to increase implantation success and ultimate survival (205, 207, 208).

In mouse pre-implantation embryos, transcripts of Cx30, 30.3, 31, 31.1, 36, 40, 43, 45 and 57 have been identified (204, 205). Connexin 43 mRNA is earliest detected in the 4-cell stage embryo with northern blotting, initiated to be transcribed with the general activation of the zygotic genome. It accumulates steadily after that until reaching a maximum at the blastocyst stage. 4-cell stage and uncompacted 8-cell stage embryos demonstrate irregular patches of cytoplasmic foci of nascent Cx43. These become punctate inter-blastomer stainings in plasma membranes with compaction (203) (211, 212). GJ assembly depends upon the mobilization of pre-existing proteins that are synthesized in the 4-cell stage (200). Connexins in the endoplasmic reticulum or endosomes before compaction are trafficked to the plasma membrane through Golgi during compaction. Uncompacted 8-cell embryos treated with trafficking inhibitors monensin and BFA demonstrate delayed acquisition of gap junctional communication and dye coupling, indicating interference with the *de novo* GJ assembly. With the increasing cell number, Cx43 becomes differentially distributed among inner and outer cells. Cx43 of outer blastomeres of late morula and TE demonstrates a zonular distribution interspersed along the apical TJ, whereas inner cells and ICM show a plaque-like punctate localization of Cx43. (203, 212, 213). Cx43 shows co-localization with cadherin-1 at cell junctions in the TE of the blastocyst (214).

Cx31 is ubiquitously expressed in cell contacts of both ICM and TE (215). Cx31.1 is detected as punctate foci on apposed cell membranes of compacting 8-cell stage embryos, whereas Cx40 demonstrates diffuse cytoplasmic immunoreactivity in 4-cell and compacted 8-cell embryos (209). Cx45 is assembled into membrane plaques at regions of blastomere apposition during compaction (216). Cx32 protein is present in zygotes, before embryonic genome activation, and throughout preimplantation development. However, Cx32 mRNA is not present in any preimplantation stage, indicating that it is inherited as an oogenetic product (217).

In order to illuminate the individual roles of connexins, experiments were designed to disrupt connexin-encoding genes by homologous recombination in embryonic stem cells (210, 216, 218). However, it is difficult to reach a conclusion regarding the individual function of a connexin isoform due to the potential for redundancy and the possibility of functional compensation of the loss by other connexins in the cells where they are co-expressed (204, 218). It is challenging to generate mouse strains with multiple null mutations for connexins by natural breeding due to the lethal phenotype of such mutations in early embryogenesis (219).

Embryonic stem cell lines that have homozygous Cx43 deletion are morphologically normal with well proliferation capacity but reduced dye-coupling ability *in vitro*, indicating decreased junctional communication. However, homozygous Cx43^(-/-)^ mutant mice develop normally through the pre-implantation period but die shortly after delivery due to swelling of the right ventricular outflow tract, interfering with pulmonary gas exchange (216, 220). Fetuses lacking both Cx32 and Cx43 survive to term but die soon after birth due to the same cardiac abnormality seen in Cx43 deficient fetuses (218). Morula that are homozygous for the Cx43 null mutation are deficient in gap junctional coupling using 6-carboxyfluorescein; however, can transmit 2’,7’-dichlorofluorescein, resembling the permeability phenotype of Cx45 (216). Thus, it was proposed that compensatory expression and up-regulation of other connexins might be sufficient to support development in embryos homozygous for the Cx43 null mutation. However, in another experiment following this hypothesis, no up-regulation of Cx31, Cx31.1, Cx40 and Cx45 were detected in mouse embryos lacking Cx43 (20). Additionally, cellular functions were not altered in coupling-deficient embryos with *Gja1* mutation. There were no significant changes in apoptosis frequency, pyruvate and glucose consumption and lactate production in the absence of Cx43 (210). Concomitantly, Cx43^(-/-)^ Cx45^(-/-)^ embryos complete peri-implantation development that is indistinguishable from that of WT, and blastocysts demonstrate a similar rate of outgrowth formation *in vitro* culture. These double mutant embryos die from cardiovascular defects, similar to Cx45^(-/-)^ embryos (219). Likewise, culturing 2-cell stage embryos in the presence of 18 a- glycyrrhetinic acid (AGA), which impairs gap junctional intercellular communication (GJIC) did not affect the mean embryo cell number, ICM/TE cell number ratio and blastocyst formation (221). However, while Cx 43 is sensitive to AGA, Cx31 and Cx45 are not (222).

In contrast, several studies demonstrated the necessity of GJIC for compaction maintenance and successful preimplantation development. Embryos from DDK female mice that naturally exhibit decreased GJIC mated with alien strain male appear phenotypically normal until the morula stage but decompact and die before blastocyst expansion. Although GJ expression increases in DDK/cross embryos, junctional plaques assemble in a disorganized pattern (213). Poor junctional communication in DDK mice is due to low cytoplasmic pH (203, 208). Similarly, when GJ antibodies that effectively block electrical coupling are injected into a cell in the 2-cell stage embryo, antibody-containing cells continue to divide but do not take part in compaction. GJ antibody injection at the 8-cell stage causes decompaction and extrusion of communication-incompetent cells and, in some cases, delayed blastocyst formation (223). Likewise, GJ antisense RNA injection to all blastomeres of 2- and 4-cell stage embryos cause a significant reduction in compaction rate. In addition, 95% of compacted 8-cell stage embryos injected with antisense RNA cannot reach the blastocyst stage (224).

In a more recent study, Shin et al. showed that Cx43 knockdown in porcine embryos significantly reduce the blastocysts’ development rate and quality of morphology due to increased reactive oxygen species (ROS) production, autophagy and apoptosis (214). In somatic cells, down-regulation of Cx43 increases ROS production, cell necrosis and apoptosis (215, 226). Increased ROS production disrupts mitochondrial membrane potential and ATP production. Therefore, Cx43 is essential in the maintenance of cellular homeostasis, mitochondrial function and quality of pre-implantation embryo (214).

## 6 Regulation of junction biogenesis

*De novo* junction biogenesis is highly dynamic and is influenced by various exogenous and intrinsic mechanisms regarding intracellular mechanobiology and intercellular junction interactions. The most extensively investigated factors regarding junction generation and maturation in the preimplantation embryo are cell-cell adhesions, asymmetric contact patterns and PKC signaling. E-cadherin adhesion and embryo compaction are prerequisites for TJ assembly during TE differentiation (160). Asymmetric contact pattern and contact-free surface alter gene expression and cellular organization pattern of blastomeres, which cause differential membrane assembly of junctional proteins within TE and ICM lineage (227). In addition, except for ZO-1α+, which is transcribed *de novo* in the blastocyst stage, the majority of TJ proteins are regulated by translational and post-translational modifications (160, 177, 178).

Four PKC isoforms (θ, δ, ι/λ, ζ) demonstrate co-localization with ZO-1 α + in apical membranes of TE, whereas remain in the cytoplasm of ICM and actively regulate blastocoel formation and TE differentiation (144). TJ proteins are differentially regulated by PKC isotypes. In isolated ICM, indolactam- and TPA- mediated PKC activation stimulates membrane assembly of ZO-2 and ZO-1 α+, and only ZO-1 α+, respectively. Both activators increase the membrane pool of PKCδ, while PKC ζ shifts to the membrane only upon TPA activation to co-localize with ZO-1 α+ (228). The influence GJIC of in the mechanodynamics of junction biogenesis in the embryo is yet to be enlightened. The association of scaffolding proteins with connexins are essential in regulating connexin turnover, channel gating and junction assembly in the plasma membrane through the organization of membrane proteins into appropriate membrane subdomains (229–233). However, in the preimplantation embryo, as explained in detail previously under the “gap junctions” title, the role of GJIC is still controversial (234). Although several studies focus on the GJ’s role in the developmental competence of the preimplantation embryo, the interaction of TJ-AJ-desmosome components with GJ structurally and functionally is yet to be discovered. **Table 1** provides a summary into determinants for junction biogenesis in the preimplantation embryo, categorized as cell-cell contact, asymmetric contact pattern, blastomere polarization, gap junctional communication, cellular metabolism, transcription factors, actomyosin skeleton and epigenetic modifications.

## 7 Conclusion

Cell-cell junctions, composed of AJs, TJs, desmosomes and GJs, are an essential component of epithelial cells for the development and maintenance of adhesion, polarized intracellular molecular architecture and mediating signaling. The preimplantation embryo undergoes various morphogenetic events, including zygotic genome activation, cleavage, compaction, polarization and asymmetric divisions that result in the formation of a blastocyst comprising an inner cell mass and outer trophectoderm layer. Epithelial characterization of the trophectoderm is critical for subsequent development, and junctional complexes are vital in generating this phenotype during early embryonic development.

**Figure 5:**
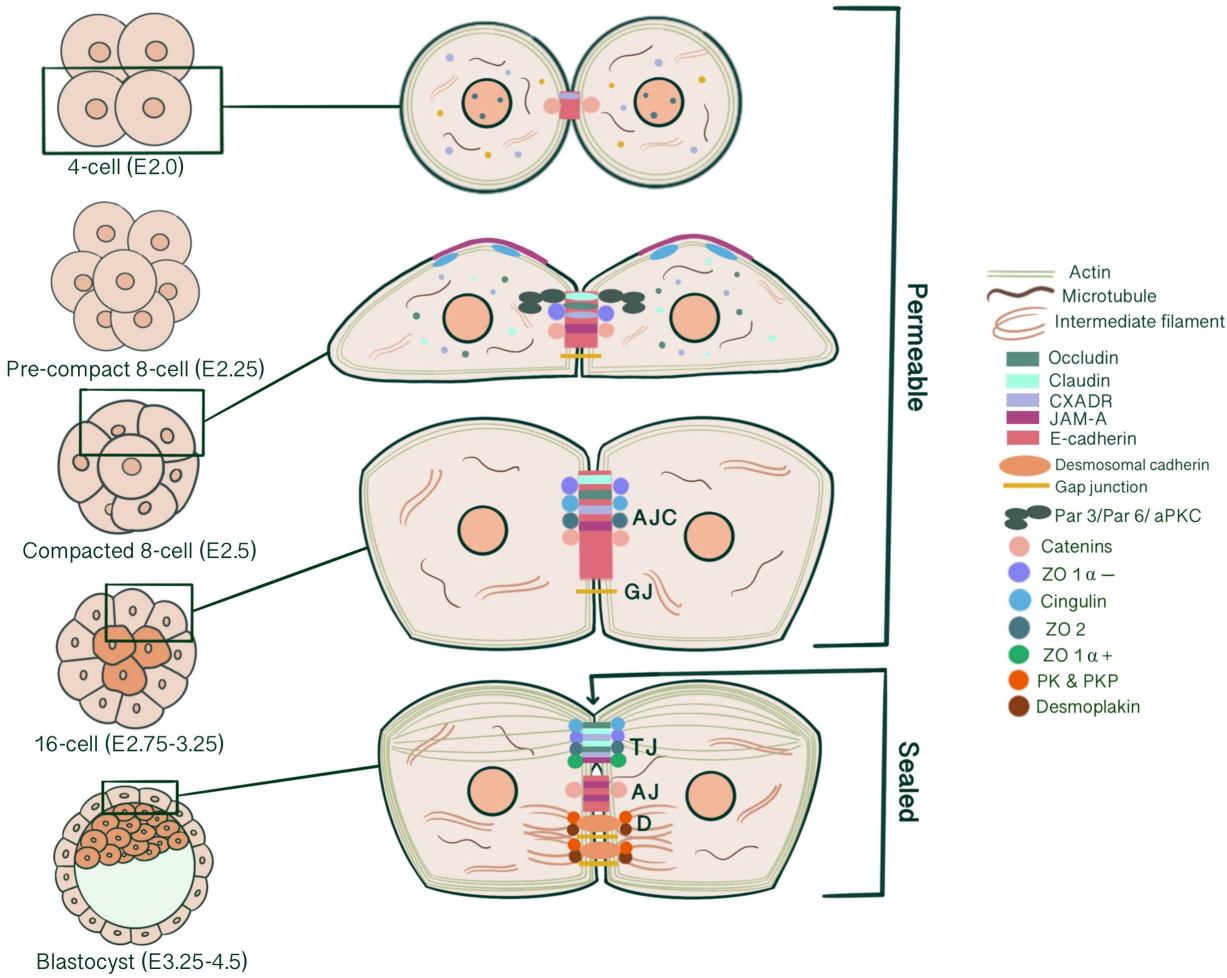
Schematic representation of apical junctional complex formation and maturation into distinct TJ, AJ, D, G starting from the 4-cell stage until the blastocyst stage, according to immunofluorescence studies from mouse pre-implantation embryos. Following the initial cleavage, until the early 8-cell stage, blastomeres are loosely connected by the E-cadherin-catenin complex localized at cell-cell contact sites. JAM family constituents assemble earlier than other TJ proteins, with CXADR starting to be detected at the 4-cell stage both in the cytoplasm and cell-cell contacts and JAM-A at the precompact 8-cell stage. With compaction, TJ constituents start to assemble in apicolateral contact regions. At the late compacted 8-cell stage, claudin and occludin start to be detected at cell membranes as punctate stains, co-localizing with ZO-1 α -, α- and β-catenin, which together form the permeable AJC. GJIC is also first evident at compacted late 8-cell stage embryos, with Cx43, 31, 31.1 and 45 localizing at membrane appositions. Cytocortical cingulin is present at the apical microvillus pole. Similarly, JAM-A intensity increases at the microvillus pole with compaction. At the 16-cell stage, ZO-2 and cingulin assemble at the contact sites for the first time, co-localizing with E-cadherin. Claudin and occludin evolve to continuous linear bands. Likewise, CXADR is also detected as continuous bands at the morula stage, with decreasing cytoplasmic intensity. Finally, at the 32-cell stage early blastocyst, ZO-1 α + localization initiates TJ/AJ segregation, forming a permeability seal. A functional TJ barrier enables the formation of the blastocoel, which is coordinated with desmosome assembly. Desmosomes start to assemble in TE at 32-cell stage regulated by desmocollin 2 expression and increase with blastocoel expansion, stabilizing TE against fluid pressure. Simultaneously to junction biogenesis, initial E-cadherin homodimerization triggers cytoskeletal reorganization. Radial arrays of actin filaments transform into circumferential actin belts below the tight junction barrier, supporting a maturing epithelial characteristic. In addition, intermediate filaments anchor to desmosomes, which appear as disc-like structures along the entire lateral border. (AJC: apical junctional complex, TJ: tight junction, AJ: adherens junction, D: desmosomes, G: gap junction).

**Figure 6:**
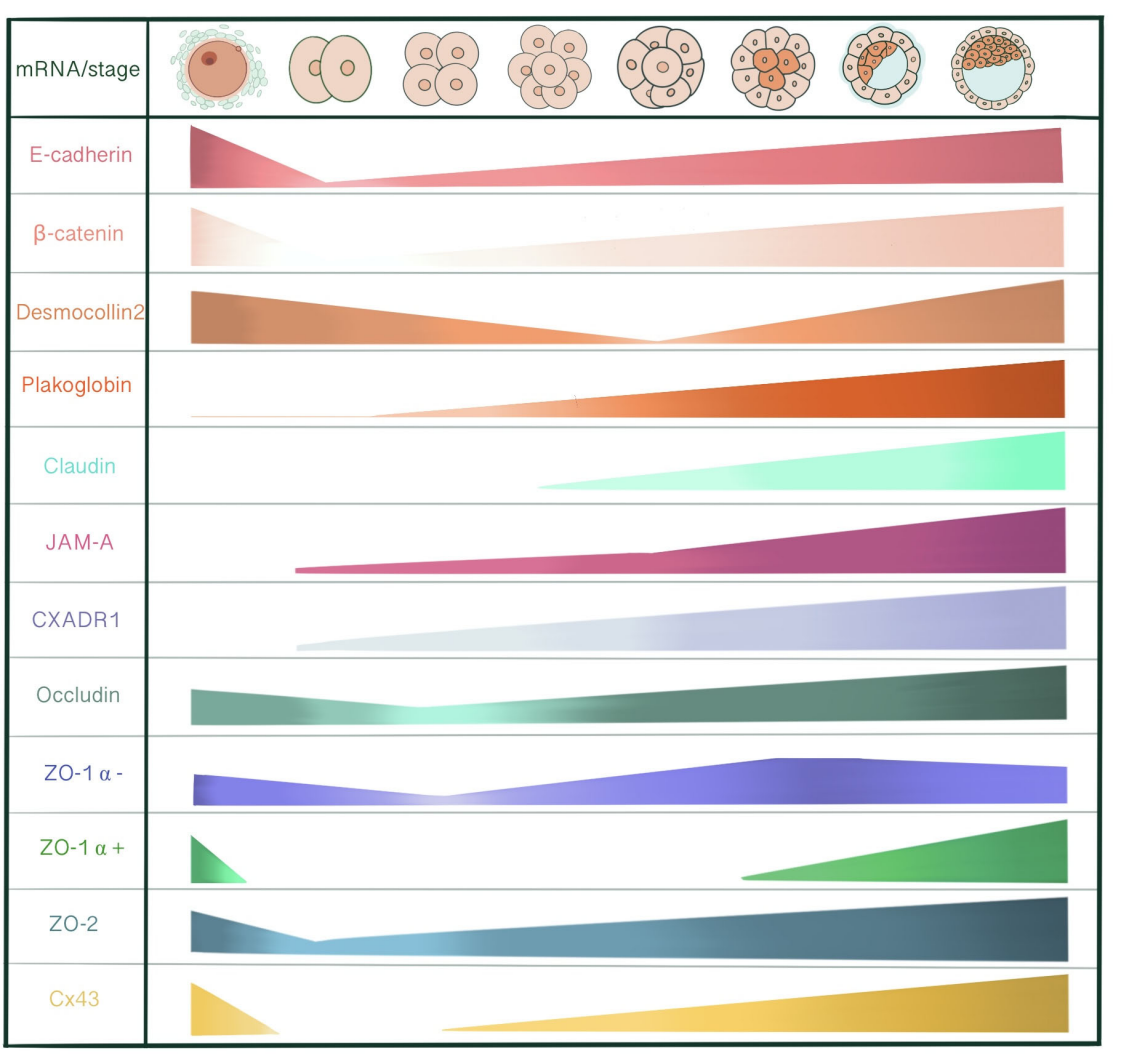
Schematic presentation of different junctional mRNA expression levels, adapted from the northern blot and RT-PCR results from mice, shown in order as cumulus-oocyte-complex, 2-cell, 4-cell, uncompacted 8-cell, compacted 8-cell, 16-cell, early blastocyst and late blastocyst stages. Junctional protein mRNA expression is temporally regulated, with distinct changes parallel to zygotic genome activation. Initially, early embryo development is directed by maternal mRNAs and proteins. Reprogramming of gene expression through the elimination of maternally deposited gene products and global activation of the zygotic genome allows maternal genome control to pass over to the zygotic genome, which predominantly occurs at the 2-cell stage in mice. Plakoglobin is very faintly detected at unfertilized eggs and the 2-cell stage, indicating a basal expression level and increases in the following developmental stages. Claudin 4, 6, 7, and 12 are detected at precompact 8-cell stage embryos. However, data is not present for mouse oocytes, zygotes and early cleavage-stage embryos. In porcine embryos, claudin 7 is present in MII oocytes as well as all developmental stages, with a significant increase at the 8-cell stage. JAM-A is not present within unfertilized eggs and starts to be detected at 2-cell stage blastomeres. However, there is no data regarding cumulus cells. CXADR1 is present in MII oocytes and increases thereafter, but there is no data regarding cumulus cells. CXADR 2 and 3 start to be detected at the morula and blastocyst stages, respectively, which is not shown in this figure. While ZO-1 α - is present in both cumulus cells and unfertilized eggs, ZO-1 α + is only present in cumulus cells. Cx43 is present within cumulus cells, oocytes and zygotes, whereas it is absent at the 2-cell stage. It increases continuously starting from the 4-cell stage onwards.

Intercellular junction assembly in preimplantation embryo demonstrates a complex spatial and temporal regulation. We provided a schematic representation of protein localization (**Figure 5**) and mRNA expression levels (**Figure 6**) of several junctional proteins according to the developmental stages in mouse preimplantation embryos. Following the initial cleavage, until the early 8-cell stage, blastomeres are loosely connected by the E-cadherin-catenin complex localized at cell-cell contact sites. E-cadherin-mediated cellular adhesion is a prerequisite for other junctional proteins to assemble into mature, functional junctions. In addition, it is essential for mediating compaction, cell fate determination by differential activation of Hippo signaling through its interaction with Amot and directing hydraulic fracturing by re-organization at cell contact sites. GJIC is first determined in late 8-cell stage embryos. Although the function of GJs in early embryonic development is still under debate, it is supported by recent studies that they are essential in maintaining cellular homeostasis, mitochondrial function and quality of the preimplantation embryo. TJ constituents, initially CXADR and JAM-A, start to assemble in apicolateral contact regions, following the order of ZO-1 α-, occludin, claudin at the compacted 8-cell stage, ZO-2 and cingulin at 16 cell stage, and ZO-1 α+ at 32-cell stage embryos, which initiates the final maturation of permeable apical junctional complex into separate AJ and TJ complexes. TJ seal formation is crucial for subsequent blastocoel formation through hydraulic fracturing and maintenance of the blastocyst integrity. Connectedly, desmosomes start to assemble in trophectoderm at the 32-cell stage regulated by desmocollin 2 expression and increase with blastocoel expansion, stabilizing trophectodermal cells against fluid pressure. Matured junctions establish a positive feedback loop that allows the blastocyst to accommodate pressure during growth. Thus, force-dependent maturation of and TJs and Ca-independent hyper-adhesive desmosomes are vital for generating a mature, viable blastocyst with structural integrity. Furthermore, the effect of AJs and TJs in modulating differential activation of the Hippo signaling pathway in inner and outer cells of morula and its impact on TE/ICM ratio indicates the importance of intercellular junctions in the formation of blastocysts with high developmental potential.

Understanding how blastomere-blastomere adhesion contributes to early embryo development may help better explain the developmental arrests of the preimplantation embryo because of growth arrest, division arrest or cell death. As junctional complexes are involved in many early developmental events either directly or indirectly, a comprehensive understanding of underlying mechanobiology regarding the developmental plasticity of preimplantation embryos, morphogenesis and cell fate specification is necessary to improve quality of preimplantation embryo development, implantation and pregnancy rates in assisted reproductive technologies. Determinants of blastocyst morphogenesis can be applied to various assessment methods and criteria; especially as potential molecular biomarkers supported by omics studies and standardized culture conditions to optimize embryo selection criteria and increase IVF success.

## Author contributions

CC analysed and interpreted of the articles in Pubmed. CC drew all the figures. EY provided substantial contribution to the design of the article. AY revised manuscript critically for important intellectual content. All authors contributed to the article and approved the submitted version.
